# The Prognosis of Single Hormone Receptor-Positive Breast Cancer Stratified by HER2 Status

**DOI:** 10.3389/fonc.2021.643956

**Published:** 2021-05-17

**Authors:** Hengqiang Zhao, Yiping Gong

**Affiliations:** Department of Breast and Thyroid Surgery, Renmin Hospital of Wuhan University, Wuhan, China

**Keywords:** breast cancer, estrogen receptor, progesterone receptor, HER2, prognosis

## Abstract

Single estrogen receptor (ER)+ and progesterone receptor (PR)+ tumors account for about10% of all breast cancers. However, the prognosis of these single hormone receptor-positive (HR+) tumor remains unclear. We aimed to investigate the characteristics of single HR+ breast tumors according to HER2 status in order to improve the treatment of patients with single HR+. Patients from the SEER program (2010-2016) were divided into ER+PR-, ER-PR+, ER+PR+ and ER-PR- molecular subtypes stratified by HER2 status. Overall survival (OS) and breast cancer-specific survival (BCSS) were compared by Kaplan–Meier curves after propensity score matching (PSM). A total of 203,406 patients were enrolled. Single ER+ and PR+ tumors account for 11.9% of the total population. For HER2- subtype, patients with ER+PR- (*n* = 16906 pairs) and ER-PR+ (*n* = 1395 pairs) had worse prognoses than those with ER+PR+ with hazard ratio (HR) and 95% confidence interval (CI) of 1.52 (1.41-1.64) and 2.25 (1.76-2.88) for OS; and 1.94 (1.76-2.14) and 2.57 (1.94-3.40) for BCSS, respectively; ER+PR- showed a better prognosis than ER-PR+ (*n* = 1394 pairs) and ER-PR- (*n* = 9626 pairs) with HR (95% CI) of 1.32 (1.06-1.65) and 1.44 (1.33-1.55) for OS, and 1.32 (1.03-1.69) and 1.46 (1.34-1.60) for BCSS, respectively; ER-PR+ had a similar prognosis relative to ER-PR- (*n* = 1395 pairs) after PSM. For HER2+ subtype, patients with ER-PR+, ER+PR-, and ER-PR- had similar OS and BCSS; ER+PR+ showed a similar prognosis compare with ER-PR+ (*n* = 535 pairs), but had better OS and BCSS than ER+PR- (*n* = 5376 pairs) and ER-PR- (*n* = 8143 pairs) after PSM. In addition, ER+PR+HER2+ showed similar OS and better BCSS compared with ER+PR+HER2- after PSM. In conclusion, single PR+ patients experienced poorer prognoses than single ER+ patients, and may be treated as ER-PR- patients in HER2- subtype. In HER2+ patients, both single ER+ and single PR+ cases showed similar prognoses compared with ER-PR- cases, and may be treated as ER-PR- patients.

## Introduction

Breast cancer is a heterogeneous malignancy that can be divided into several molecular subtypes according to estrogen receptor (ER), progesterone receptor (PR), and human epidermal growth factor 2 (HER2). ER and PR are termed as hormone receptor (HR). The four main molecular subtypes are luminal A, luminal B, HER2+, and triple-negative breast cancer. This classification provides a good biomarker for prognosis and basis for targeted therapies.

Single ER+ and PR+ subtypes accounted for about 10% of all molecular subtypes of breast cancer (1). Some studies found that there were no differences in prognosis between ER+PR- and ER-PR+ patients (2, 3). Others found that ER-PR+ patients had worse prognosis compared with ER+PR- cases (1, 4). Research from the National Cancer Database and the Surveillance, Epidemiology, and End Results (SEER) program showed the same conclusion that single HR+ tumors had worse prognosis than ER+PR+ tumors, and ER-PR+ subtype had similar prognosis relative to ER-PR- (5, 6). However, HER2 status, a vital predictor of breast cancer prognosis, was not included for analysis in these studies. Recently, a single-center study found that the clinicopathologic characteristics and prognosis were similar between ER+PR+, ER-PR+, and ER+PR- in HER2+ patients (7). Due to the limited sample size of single HR+ cases and the imbalance between groups, they obtained a meaningless and wide confidence interval and this may weaken the reliability of the results (7). To our knowledge, studies on the prognosis of single ER+ and PR+ stratified by HER2 status are very limited.

In order to discern and treat patients with single HR+ better, we compared the prognosis between single ER+ and PR+ tumors, and compared them with the other molecular subtypes stratified by HER2 status. Unlike previous studies, we for the first time balanced the variables between groups using propensity score matching (PSM), and we found some novel results of the prognosis of single HR+ tumors after introducing HER2.

## Patients and Methods

### Ethics Statement

The study population were obtained from SEER database from 2010 to 2016 (SEERStat user name: 10561-Nov2019). This study was granted an exemption from institutional review board approval for its deidentified information in a public database.

### Study Population

ER and PR status were determined by immunohistochemistry. If 1% or greater cells stain positive, the test results are considered positive. HER2 status was available from 2010 in SEER database, we thus enroll patients from 2010. The following clinicopathologic characteristics were extracted from the database: patient age, year of diagnosis, race, marital status, insurance, pathological types, tumor grade, tumor stage, T/N/M stage, number of metastatic axillary lymph nodes, surgery, radiation, chemotherapy, overall survival (OS), breast cancer-specific survival (BCSS), and survival months. Marital status comprises single, married, divorced/separated/widowed (DSW) and other. The pathology was categorized into invasive ductal carcinoma, invasive lobular carcinoma, and invasive ductal and lobular carcinoma. Poorly differentiated and anaplastic histology were defined as grade III. Tumor stage and T/N/M stage was based on American Joint Committee on Cancer (AJCC) stage of the 6th edition. Surgery was categorized into partial mastectomy, total mastectomy, and modified radical mastectomy. Only female patients with one primary tumor, positive histology, and age ≥ 18 years were enrolled. Data with unknown information were excluded.

### Statistical Analysis

Category variables were analyzed by χ^2^ test. Age and year of diagnosis were analyzed with Mann–Whitney *U* test. The number of metastatic axillary lymph nodes was compared by Student’s *t* test. OS was defined as the duration between the date of initial diagnosis to death from any causes or last follow up. BCSS was defined as the time from diagnosis to death from breast cancer. The Kaplan–Meier curves of OS and BCSS were analyzed by log-rank test. Multivariable Cox proportional hazards regression was established to estimate hazard ratio (HR) and a 95% confidence interval (CI) for OS and BCSS.

To minimize the imbalance of the variables between groups, PSM was performed using R software (ver. 3.3.3, https://www.r-project.org/) of package ‘MatchIt’. Age, year of diagnosis, race, marital status, insurance, histology, grade, tumor stage, T/N/M stage, metastatic axillary lymph nodes, surgery, radiation, and chemotherapy were matched between groups. One-to-one matching with a caliper of 0.1 was used to balance the demographic, pathologic and treatment covariates as previously described (8). Other statistical analyses were performed with Stata/MP (ver. 14.2, StataCorp., College Station, TX), and GraphPad Prism (ver. 7.0, GraphPad Software, Inc). A two-sided *P* value < 0.05 was considered statistically different.

## Results

### Clinicopathologic Characteristics of the Study Population

The flowchart of selection process was shown in **Supplementary Figure 1**. A total of 203406 patients were included, including 133662 patients (65.7%) for ER+PR+HER2-, 16906 (8.3%) for ER+PR-HER2-, 1395 (0.7%) for ER-PR+HER2-, 21439 (10.5%) for ER-PR-HER2-; and 15646 (7.7%) for ER+PR+HER2+, 5381 (2.6%) for ER+PR-HER2+, 537 (0.3%) for ER-PR+HER2+, and 8440 (4.1%) for ER-PR-HER2+. The median follow-up duration of the study population was 35 months (range: 1-83 months). The clinicopathologic characteristics of each subtype were summarized in **Table 1**.

**Table 1:** Clinicopathologic characteristics of the study population (*n* = 203,406).

Category	HER2-				HER2+			
	ER-PR+ (*n* = 1395)	ER+PR- (*n* = 16906)	ER-PR- (*n* = 21439)	ER+PR+ (*n* = 133662)	ER-PR+ (*n* = 537)	ER+PR- (*n* = 5381)	ER-PR- (*n* = 8440)	ER+PR+ (*n* = 15646)
Age (year)	57 (48-67)	62 (54-70)	55 (47-65)	61 (51-69)	56 (47-65)	58 (51-66)	56 (48-65)	56 (46-65)
Year of diagnosis	2013 (2011-2015)	2013 (2011-2015)	2013 (2011-2015)	2013 (2011-2015)	2013 (2012-2015)	2013 (2011-2015)	2013 (2011-2015)	2013 (2011-2015)
Race								
White	1021 (73.2)	13095 (77.5)	15403 (71.8)	109651 (82.0)	395 (73.6)	4079 (75.8)	6055 (71.7)	12144 (77.6)
Black	270 (19.4)	2196 (13.0)	4366 (20.4)	10889 (8.1)	70 (13.0)	645 (12.0)	1187 (14.1)	1736 (11.1)
Other	104 (7.5)	1615 (9.6)	1670 (7.8)	13122 (9.8)	72 (13.4)	657 (12.2)	1198 (14.2)	1766 (11.3)
Insurance								
Uninsured	25 (1.8)	217 (1.3)	417 (1.9)	1675 (1.3)	10 (1.9)	88 (1.6)	152 (1.8)	263 (1.7)
Insured	1370 (98.2)	16689 (98.7)	21022 (98.1)	131987 (98.7)	527 (98.1)	5293 (98.4)	8288 (98.2)	15383 (98.3)
Marital status								
Single	231 (16.6)	2413 (14.3)	3571 (16.7)	18758 (14.0)	71 (13.2)	837 (15.6)	1290 (15.3)	2635 (16.8)
Married	796 (57.1)	9424 (55.7)	11941 (55.7)	78702 (58.9)	322 (60.0)	3122 (58.0)	4997 (59.2)	9298 (59.4)
DSW	308 (22.1)	4369 (25.8)	4944 (23.1)	30837 (23.1)	118 (22.0)	1203 (22.4)	1796 (21.3)	3111 (19.9)
Other	60 (4.3)	700 (4.1)	983 (4.6)	5365 (4.0)	26 (4.8)	219 (4.1)	357 (4.2)	602 (3.8)
Grade								
I	21 (1.5)	3415 (20.2)	308 (1.4)	40471 (30.3)	6 (1.1)	236 (4.4)	120 (1.4)	1123 (7.2)
II	218 (15.6)	7147 (42.3)	3476 (16.2)	70474 (52.7)	125 (23.3)	1984 (36.9)	1924 (22.8)	6860 (43.8)
III-IV	1156 (82.9)	6344 (37.5)	17655 (82.3)	22717 (17.0)	406 (75.6)	3161 (58.7)	6396 (75.8)	7663 (49.0)
Pathology								
IDC	1350 (96.8)	13397 (79.2)	20920 (97.6)	107194 (80.2)	522 (97.2)	5015 (93.2)	8271 (98.0)	14222 (90.9)
ILC	19 (1.4)	2501 (14.8)	222 (1.0)	16067 (12.0)	2 (0.4)	175 (3.3)	70 (0.8)	657 (4.2)
IDLC	26 (1.9)	1008 (6.0)	297 (1.4)	10401 (7.8)	13 (2.4)	191 (3.5)	99 (1.2)	767 (4.9)
Stage								
I	531 (38.1)	8453 (50.0)	8414 (39.2)	78791 (58.9)	194 (36.1)	2324 (43.2)	3232 (38.3)	6913 (44.2)
II	681 (48.8)	6107 (36.1)	9633 (44.9)	41854 (31.3)	243 (45.3)	2161 (40.2)	3480 (41.2)	6322 (40.4)
III	161 (11.5)	2090 (12.4)	2985 (13.9)	11891 (8.9)	88 (16.4)	754 (14.0)	1481 (17.5)	2124 (13.6)
IV	22 (1.6)	256 (1.5)	407 (1.9)	1126 (0.8)	12 (2.2)	142 (2.6)	247 (2.9)	287 (1.8)
Tumor								
T1	633 (45.4)	9704 (57.4)	9862 (46.0)	90610 (67.8)	252 (46.9)	2822 (52.4)	4019 (47.6)	8337 (53.3)
T2	621 (44.5)	5663 (33.5)	9159 (42.7)	35490 (26.6)	217 (40.4)	1993 (37.0)	3296 (39.1)	5879 (37.6)
T3	89 (6.4)	1135 (6.7)	1625 (7.6)	5869 (4.4)	40 (7.4)	391 (7.3)	727 (8.6)	969 (6.2)
T4	52 (3.7)	404 (2.4)	793 (3.7)	1693 (1.3)	28 (5.2)	175 (3.3)	398 (4.7)	461 (2.9)
Node								
N0	954 (68.4)	11583 (68.5)	14458 (67.4)	94487 (70.7)	320 (59.6)	3398 (63.1)	5069 (60.1)	9799 (62.6)
N1	328 (23.5)	3682 (21.8)	4732 (22.1)	29698 (22.2)	156 (29.1)	1373 (25.5)	2230 (26.4)	4190 (26.8)
N2	62 (4.4)	1014 (6.0)	1355 (6.3)	6467 (4.8)	42 (7.8)	363 (6.7)	653 (7.7)	1087 (6.9)
N3	51 (3.7)	627 (3.7)	894 (4.2)	3010 (2.3)	19 (3.5)	247 (4.6)	488 (5.8)	570 (3.6)
ALNM	1.04 ± 3.39	1.18 ± 3.38	1.19 ± 3.39	0.92 ± 2.68	1.33 ± 3.29	1.23 ± 3.17	1.52 ± 3.88	1.24 ± 3.19
Metastasis								
M0	1373 (98.4)	16650 (98.5)	21032 (98.1)	132536 (99.2)	525 (97.8)	5239 (97.4)	8193 (97.1)	15359 (98.2)
M1	22 (1.6)	256 (1.5)	407 (1.9)	1126 (0.8)	12 (2.2)	142 (2.6)	247 (2.9)	287 (1.8)
Surgery								
PM	785 (56.3)	9891 (58.5)	11441 (53.4)	84530 (63.2)	240 (44.7)	2493 (46.3)	3618 (42.9)	8043 (51.4)
TM	370 (26.5)	4192 (24.8)	5712 (26.6)	32232 (24.1)	180 (33.5)	1719 (31.9)	2702 (32.0)	4605 (29.4)
MRM	240 (17.2)	2823 (16.7)	4286 (20.0)	16900 (12.6)	117 (21.8)	1169 (21.7)	2120 (25.1)	2998 (19.2)
Radiation								
No	561 (40.2)	6687 (39.6)	9307 (43.4)	50080 (37.5)	246 (45.8)	2610 (48.5)	4174 (49.5)	6909 (44.2)
Yes	834 (59.8)	10219 (60.4)	12132 (56.6)	83582 (62.5)	291 (54.2)	2771 (51.5)	4266 (50.5)	8737 (55.8)
Chemotherapy								
No	351 (25.2)	9122 (54.0)	4740 (22.1)	95852 (71.7)	105 (19.6)	1335 (24.8)	1768 (20.9)	3792 (24.2)
Yes	1044 (74.8)	7784 (46.0)	16699 (77.9)	37810 (28.3)	432 (80.4)	4046 (75.2)	6672 (79.1)	11854 (75.8)

Data were expressed as number (%), median (interquartile), or mean ± standard deviation. DSW, divorced/separated/widowed; IDC, invasive ductal carcinoma; ILC, invasive lobular carcinoma; IDLC, invasive ductal and lobular carcinoma; ER, estrogen receptor; PR, progesterone receptor; HER2, human epidermal growth factor receptor 2; ALNM, axillary lymph nodes metastasis; PM, partial mastectomy; TM, total mastectomy; MRM; modified radical mastectomy.

### Predictors for OS and BCSS by Multivariate Cox Regression Analysis

Compared with ER+PR+HER2-, patients with ER+PR-HER2-, ER-PR+HER2-, and ER-PR-HER2- were associated with compromised OS with HR (95% CI) of 1.67 (1.58-1.77), 2.36 (2.02-2.75), and 2.72 (2.59-2.87), respectively. In addition, ER+PR-HER2+ and ER-PR-HER2+ were associated with compromised OS compared with ER+PR+HER2- with HR (95% CI) of 1.20 (1.09-1.34) and 1.27 (1.17-1.38), respectively. However, patients with ER+PR+HER2+ showed marginally better OS than those with ER+PR+HER2-with HR (95% CI) of 0.93 (0.86-1.01). No significant difference in OS was observed between patients with ER+PR+HER2- and ER-PR+HER2+ (**Table 2**). BCSS showed the same trend as OS. In addition, patients with ER+PR+HER2+ showed improved BCSS compared with ER+PR+HER2- cases with HR (95% CI) of 0.89 (0.80-0.99) (**Supplementary Table 1**).

**Table 2:** Overall survival by multivariate Cox proportional analysis.

Category	HR (95% CI)	*P-*value
Age	1.038 (1.036-1.039)	<0.001
Year of diagnosis	0.98 (0.96-0.99)	<0.001
Race (vs White)		
Black	1.23 (1.17-1.29)	<0.001
Other	0.71 (0.66-0.77)	<0.001
Insurance (vs uninsured)		
Insured	0.82 (0.71-0.93)	0.003
Marital status (vs single)		
Married	0.73 (0.69-0.77)	<0.001
Divorced/separated/widowed	1.01 (0.96-1.07)	0.653
Other	0.84 (0.76-0.92)	<0.001
Grade (vs I)		
II	1.24 (1.16-1.32)	<0.001
III-IV	1.88 (1.76-2.01)	<0.001
Pathology (vs invasive ductal carcinoma)		
Invasive lobular carcinoma	0.86 (0.80-0.92)	<0.001
Invasive ductal and lobular carcinoma	0.91 (0.84-0.98)	0.015
Tumor (vs T1)		
T2	1.72 (1.65-1.80)	<0.001
T3	2.53 (2.37-2.70)	<0.001
T4	3.15 (2.92-3.40)	<0.001
Node (vs N0)		
N1	1.72 (1.65-1.80)	<0.001
N2	2.53 (2.37-2.70)	<0.001
N3	3.15 (2.92-3.40)	<0.001
Axillary lymph nodes metastasis	1.03 (1.02-1.04)	<0.001
Metastasis (vs M0)		
M1	2.90 (2.70-3.11)	<0.001
Molecular subtypes (vs ER+PR+HER2-)		
ER+PR-HER2-	1.67 (1.58-1.77)	<0.001
ER-PR+HER2-	2.36 (2.02-2.75)	<0.001
ER-PR-HER2-	2.72 (2.59-2.87)	<0.001
ER+PR+HER2+	0.93 (0.86-1.01)	0.071
ER+PR-HER2+	1.20 (1.09-1.34)	<0.001
ER-PR+HER2+	1.04 (0.74-1.46)	0.805
ER-PR-HER2+	1.27 (1.17-1.38)	<0.001
Surgery (vs partial mastectomy)		
Total mastectomy	1.02 (0.96-1.07)	0.584
Modified radical mastectomy	1.22 (1.16-1.28)	<0.001
Radiation (vs no)		
Yes	0.66 (0.63-0.68)	<0.001
Chemotherapy (vs no)		
Yes	0.73 (0.70-0.76)	<0.001

HR, hazard ratio; CI, confidence interval; ER, estrogen receptor; PR, progesterone receptor.

### Kaplan–Meier Curves of OS and BCSS Before PSM

**Figure 1:**
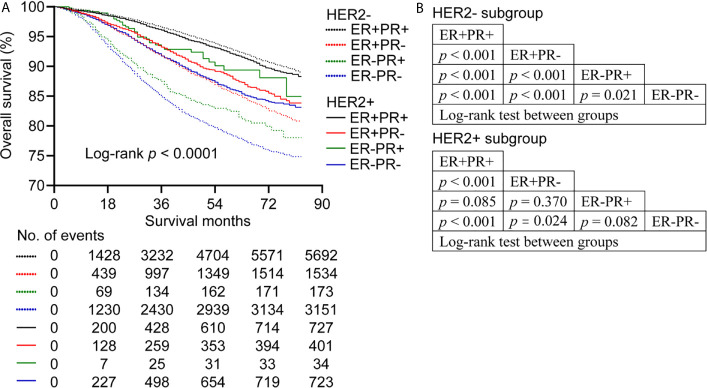
Overall survival of patients stratified by estrogen receptor (ER), progesterone receptor (PR) and human epidermal growth factor Receptor 2 (HER2) status before propensity score matching. **(A)** Kaplan-Meier survival curves of overall survival. **(B)** League table of comparison by log-rank test.

For HER2- subtype, ER+PR+ had better OS compared with ER+PR-, ER-PR+, and ER-PR- (log-rank *p* < 0.001 for all). In addition, ER+PR- showed better OS than ER-PR+ and ER-PR- (log-rank *p* < 0.001 for both). Additionally, ER-PR+ showed better OS than ER-PR- (log-rank *p* = 0.021) before PSM (**Figures 1A, B**).

For HER2+ subgroup, ER+PR+ showed better OS than ER+PR- and ER-PR- (log-rank *p* < 0.001 for both), while ER+PR+ had similar OS relative to ER-PR+ (log-rank *p* = 0.085). In addition, ER+PR- showed better OS than ER-PR- (log-rank *p* = 0.024). However, no significant difference in OS was observed between ER+PR- and ER-PR+ (log-rank *p* = 0.370). What’s more, ER-PR+ showed similar OS compared with ER-PR- (log-rank *p* = 0.082) (**Figures 1A, B**). BCSS showed the same trend as OS. In addition, ER-PR+ patients showed better BCSS than ER-PR- patients before PSM (log-rank *p* = 0.045) (**Supplementary Figure 2**).

### Kaplan–Meier Curves of OS and BCSS After PSM

To minimize the imbalance of baseline clinicopathologic characteristics between groups, PSM was performed. The absolute values of standardized differences of the matched variables were < 0.1, indicating that the variables were well balanced between groups after matching. In addition, the statistical differences in the baseline characteristics between groups were reduced after PSM.

**Figure 2:**
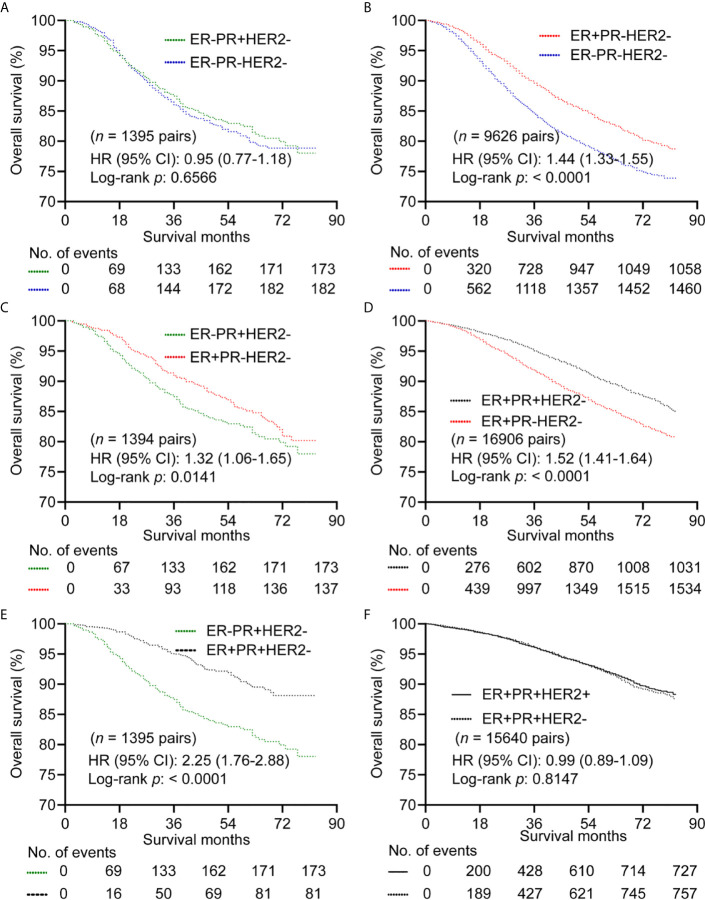
Kaplan–Meier curves of overall survival after propensity score matching in HER2- subgroup. **(A)** Comparison between ER-PR+ and ER-PR-. **(B)** Comparison between ER+PR- and ER-PR-. **(C)** Comparison between ER-PR+ and ER+PR-. **(D)** Comparison between ER+PR+ and ER+PR-. **(E)** Comparison between ER+PR+ and ER-PR+. **(F)** Comparison between ER+PR+HER2- and ER+PR+HER2+. ER, estrogen receptor; PR, progesterone receptor; HER2, human epidermal growth factor Receptor 2.

For HER2- subgroup, there were no differences in OS between ER-PR+ and ER-PR- with HR (95% CI) of 0.95 (0.77-1.18) in a matched cohort of 1395 paired cases (**Figure 2A** and **Supplementary Table 2**). However, ER+PR- showed better OS than ER-PR- with HR (95% CI) of 1.44 (1.31-1.55) in a matched cohort of 9626 paired cases (**Figure 2B** and **Supplementary Table 3**). Patients with ER+PR- showed better OS than patients with ER-PR+ (*n* = 1394 pairs) with HR (95% CI) of 1.32 (1.06-1.65) (**Figure 2C** and **Supplementary Table 4**). ER+PR+ showed better OS than ER+PR- with HR (95% CI) of 1.52 (1.41-1.64) (*n* = 16906 pairs) (**Figure 2D**; **Supplementary Table 5**), and better OS than ER-PR+ (*n* = 1395 pairs) with HR (95% CI) of 2.25 (1.76-2.88) (**Figure 2E** and **Supplementary Table 6**). We further found that there was no difference in OS between ER+PR+HER2+ and ER+PR+HER2-with HR (95% CI) of 0.99 (0.89-1.09) (*n* = 15640 pairs) (**Figure 2F** and **Supplementary Table 7**). BCSS showed the same trend as OS. However, patients with ER+PR+HER2+ predicted better BCSS than those with ER+PR+HER2- with HR (95% CI) of 0.86 (0.76-0.98) (**Supplementary Figure 3**).

**Figure 3:**
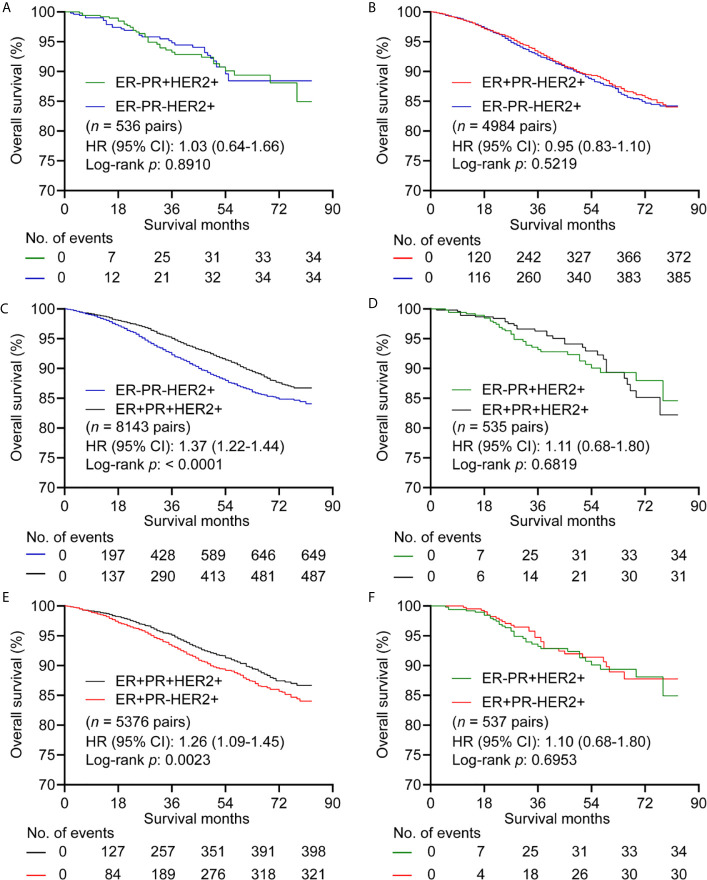
Kaplan–Meier curves of overall survival in HER2+ subgroup after propensity score matching. **(A)** Comparison between ER-PR+ and ER-PR-. **(B)** Comparison between ER+PR- and ER-PR-. **(C)** Comparison between ER-PR- and ER+PR+. **(D)** Comparison between ER+PR+ and ER-PR+. **(E)** Comparison between ER+PR+ and ER+PR-. **(F)** Comparison between ER+PR- and ER-PR+. ER, estrogen receptor; PR, progesterone receptor; HER2, human epidermal growth factor Receptor 2.

For HER2+ subgroup, patients with ER-PR- showed similar OS compared with ER-PR+ (*n* = 536 pairs) (**Figure 3A** and **Supplementary Table 8**), and ER+PR- (*n* = 4984 pairs) (**Figure 3B** and **Supplementary Table 9**). ER+PR+ showed better OS than ER-PR- with HR (95% CI) of 1.37 (1.22-1.44) (*n* = 8143 pairs) (**Figure 3C** and **Supplementary Table 10**). No significant differences in OS were observed between patient with ER+PR+ and ER-PR+ (*n* = 535 pairs) (**Figure 3D** and **Supplementary Table 11**). Patients with ER+PR+ had better OS than patients with ER+PR- with HR (95% CI) of 1.26 (1.09-1.45) (*n* = 5376 pairs) (**Figure 3E** and **Supplementary Table 12**). There was no significant difference in OS between patients with ER+PR- and ER-PR+ with HR (95% CI) of 1.10 (0.68-1.80) (*n* = 537 pairs) (**Figure 3F** and **Supplementary Table 13**). BCSS showed the same trend as OS (**Supplementary Figure 4**).

## Discussion

In this study, we found that single HR+ showed different effect on the prognosis of HER2- and HER2+ breast tumors. ER+PR- had better prognosis than ER-PR+ in HER2- cases. However, ER+PR- showed similar prognosis compared with ER-PR+ in HER2+ cases. In addition, both ER+PR- and ER-PR+ cases had higher overall mortality than ER+PR+ patients in HER2- subtype. For HER2+ subtype, ER-PR+ showed similar prognosis compared with ER+PR+. ER+PR+HER2+ patients had similar OS and better BCSS relative to those with ER+PR+HER2-.

A recent study found that ER+PR- and ER-PR+ had similar disease-free survival and OS compared with ER-PR- in both HER2+ and HER2- subgroups (7). However, we found that ER+PR- showed better OS and BCSS than ER-PR- in HER2- subgroup. This inconsistence might result from the sample size, statistical methods, and the adjusted variables. In addition, this study failed to compared the survival difference between ER+PR- and ER-PR+ (7). We found that there were no differences in the prognoses between ER+PR- and ER-PR+ and ER-PR- after PSM in HER2+ subgroup. This indicated that patients with single ER+ or PR+ may gain limited survival benefit from endocrine therapy when facing HER2+. For the limited sample size, they failed to fully investigate the survival differences of ER+PR+ (*n* = 518) and ER-PR+ (*n* = 30) and ER+PR- (*n* = 159) in HER2+ subtype. We found that patients with ER+PR+ (*n* = 15646) showed similar prognosis relative to those with ER-PR+ (*n* = 537), but had better prognosis than patients with ER+PR- (*n* = 5381) and ER-PR- (*n* = 8840) in HER2+ subtype after PSM.

The clinicopathologic characteristics were highly consistent between ER-PR+HER2+ and ER-PR-HER2+. However, patients with ER+PR-HER2+ were older, had lower tumor grade and tumor stage compared with ER-PR-HER2+. It seemed that patients with ER-PR+HER2+ were more likely to gain survival benefit from endocrine therapy or HER2-targeted therapy than those with ER+PR-HER2+. It indicated that PR levels may reflect growth factor activity within a tumor. Low or absent PR expression in some tumors indicated high HER2 activity. The increased growth factor signaling may reduce the ability of tamoxifen to act as an antagonist, resulting in selective ER modulator resistance (9).

Unlike the role of ER+PR- in HER2+ tumors, patients with ER+PR- showed better prognoses relative to those with ER-PR+ and ER-PR- in HER2- subtype after PSM. We found that ER-PR+HER2- showed similar prognosis compared with ER-PR-HER2-. In addition, ER+PR+HER2- showed better OS and BCSS than ER+PR-HER2-. Considering the survival differences between ER+PR- and ER-PR- in HER2+ and HER2- subtypes, we can speculate that there existed a signaling crosstalk between ER/PR and HER2. Growth factor can directly modulate ER activity *via* phosphorylation of ER itself or *via* phosphorylation of coregulators, it also downregulated PR levels independent of ER levels or activity (9). The proportion of ER+PR- and ER-PR+ tumors was 11.9% of in this study. ER and PR status can change during breast cancer development (10, 11). Therefore, the conversion from ER+PR+ to single ER+ or PR+, or to ER-PR- may indicate disease progression.

The proportion of ER-PR+ phenotype represents 1.0% of the total patients in our study. A study re-evaluated 43 of 2432 (1.8%) patients reported as ER-PR+ in a reference laboratory. However, none of the cases were the initial ER-PR+ (12). Another study re-evaluated 27 of 9844 (0.3%) patients which were initially diagnosed with ER-PR+ breast carcinoma, and 7 patients remained ER-PR+ (13). However, another study found that breast carcinoma of ER-PR+ existed, although rare (1.1% of all phenotypes), and had distinct clinicopathologic characteristics (14). The status of ER-PR+ should be evaluated carefully to avoid technical artifacts (15).

Contrary to our conventional thought that ER+PR+HER2- predicted the best survival, and a low risk of local or regional recurrence (16, 17), we found that patients with ER+PR+HER2+ showed similar OS, and even better BCSS relative to those with ER+PR+HER2-. A previous study showed that HR+HER2+ subtype experienced the better BCSS than those with HR+HER2- for stage IV breast cancer (16). The introduction of trastuzumab therapy may account for the improved prognosis of HER2+ patients. Trastuzumab plus adjuvant chemotherapy was associated with a 33% reduction in the risk of mortality compared with chemotherapy alone for operable HER2+ breast cancer (18). In CLEOPATRA trial, dual inhibition of HER2 signaling with pertuzumab and trastuzumab significantly improved OS compared with trastuzumab alone for HER2+ metastatic breast cancer (median OS: 56.5 months vs 40.8 months) (19). With the development of HER2 inhibitors, patients with ER+PR+HER2+ may gain better prognoses than those with ER+PR+HER2-.

The limitations of this study must be acknowledged. First, the selection bias was evitable for the retrospective design. However, the statistical differences in baseline variables were significantly reduced after performing PSM, which may improve the reliability of the results. In addition, the endocrine and HER2 targeted therapies were not recorded in the database. Therefore, they were not included for analysis. Additionally, the exact expression levels of ER and PR were unavailable. We can’t re-evaluate single ER+ and PR+ status.

In conclusion, this is the largest known study investigating the prognosis of breast cancer stratified by ER, PR and HER2 status. We found that single ER+ and PR+ subtypes showed different roles in the prognosis of breast cancer according to HER2 status. For HER2- subgroup, ER+PR+ showed the best prognosis, followed by ER+PR-, and worst for ER-PR+ and ER-PR- subtypes. For HER2+ subgroup, ER+PR+ showed similar prognosis relative to ER-PR+, but better prognosis than ER+PR- and ER-PR- subtypes. HER2 status didn’t decrease the survival of patients with ER+PR+. Although we strictly included cases and balanced the variables between groups with a large population, the results should be cautiously interpreted when dealing with single ER+ or PR+ breast cancers.

## Data Availability Statement

The original contributions presented in the study are included in the article/**Supplementary Material**. Further inquiries can be directed to the corresponding author.

## Author Contributions

HZ: conception, data acquisition. HZ and YG: data analysis and drafting the article. HZ and YG: revised it critically for important intellectual content. HZ: investigation, project administration, and supervision. All authors contributed to the article and approved the submitted version.

## Funding

This work was supported by the Fundamental Research Funds for the Central Universities (grant number 2042020kf0063).

## Conflict of Interest

The authors declare that the research was conducted in the absence of any commercial or financial relationships that could be construed as a potential conflict of interest.
