# Supplementary material for: The Prognosis of Single Hormone Receptor-Positive Breast Cancer Stratified by HER2 Status
Source: Front Oncol. 2021 May 17;11:643956. doi: 10.3389/fonc.2021.643956 (PMC8165305; doi:10.3389/fonc.2021.643956)
Supplement: Supplementary file 1 [file DataSheet_1.docx]

**Supplementary data**


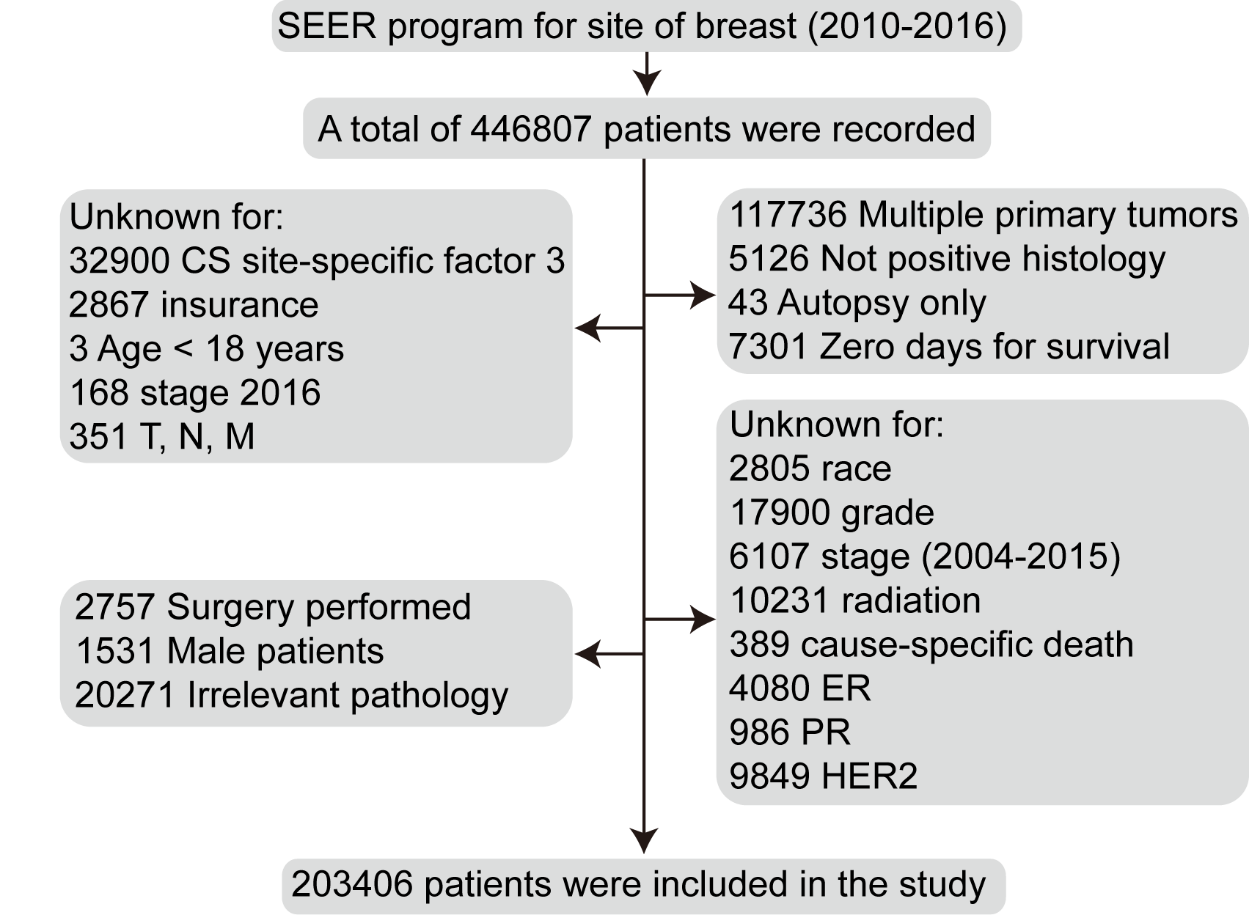


**Supplementary Fig. 1** The flowchart of the selection process


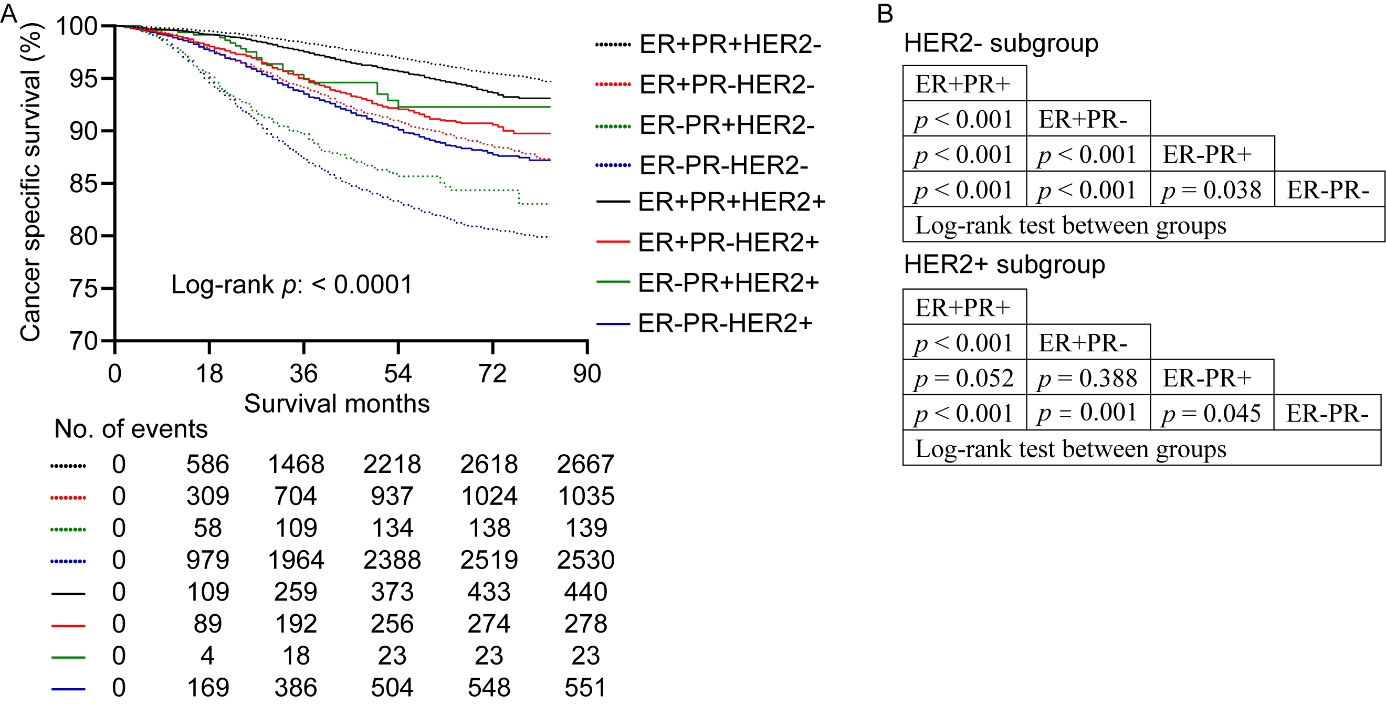


**Supplementary Fig. 2** Breast cancer-specific survival of patients stratified by estrogen receptor (ER), progesterone receptor (PR) and human epidermal growth factor Receptor 2 (HER2) status before propensity score matching. (A) Kaplan-Meier survival curves of breast cancer-specific survival. (B) League table of comparison by log-rank test.


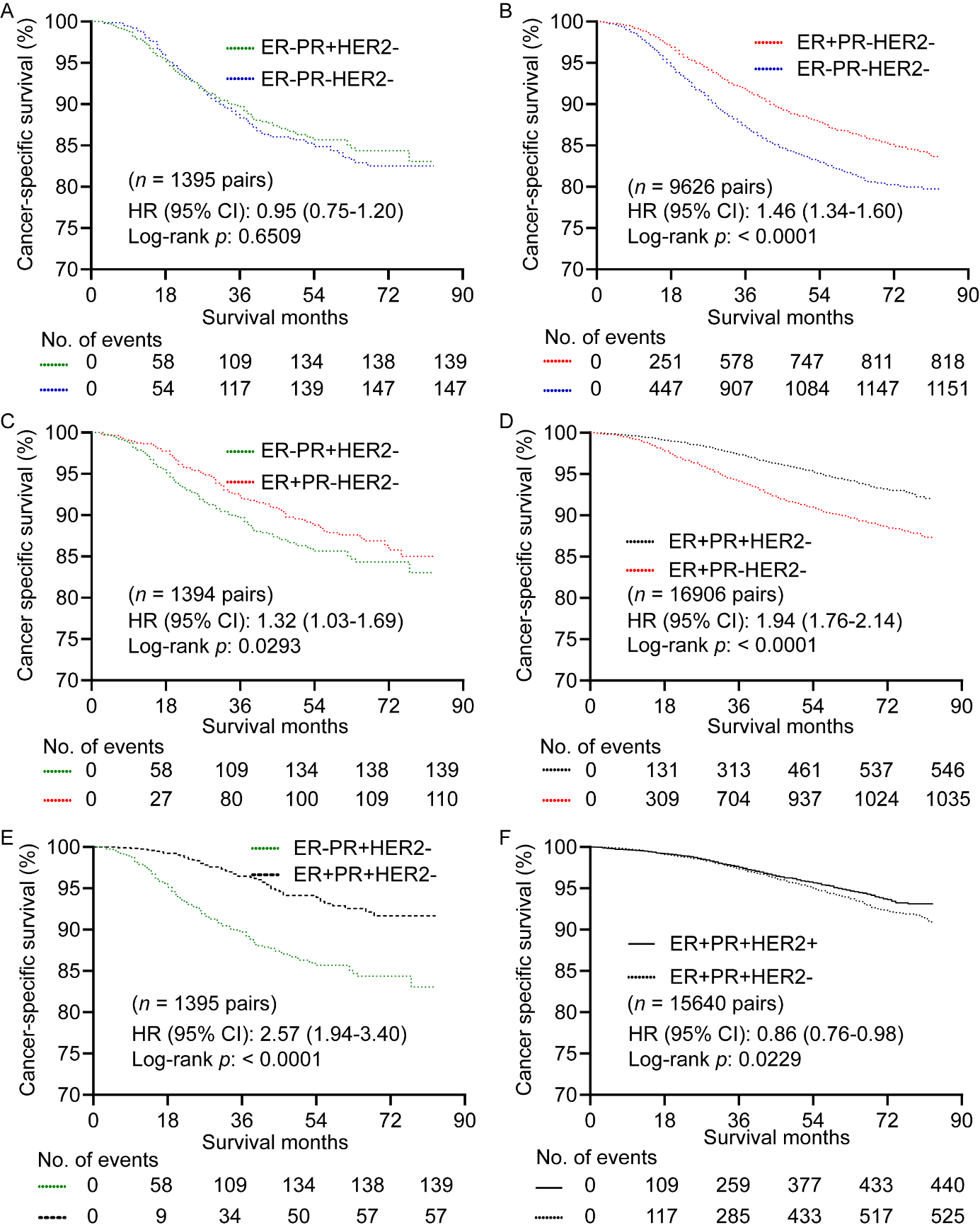


**Supplementary Fig. 3** Kaplan–Meier curves of breast cancer-specific survival after propensity score matching in HER2- subgroup. (A) Comparison between ER-PR+ and ER-PR-. (B) Comparison between ER+PR- and ER-PR-. (C) Comparison between ER-PR+ and ER+PR-. (D) Comparison between ER+PR+ and ER+PR-. (E) Comparison between ER+PR+ and ER-PR+. (F) Comparison between ER+PR+HER2- and ER+PR+HER2+. ER, estrogen receptor; PR, progesterone receptor; HER2, human epidermal growth factor Receptor 2.


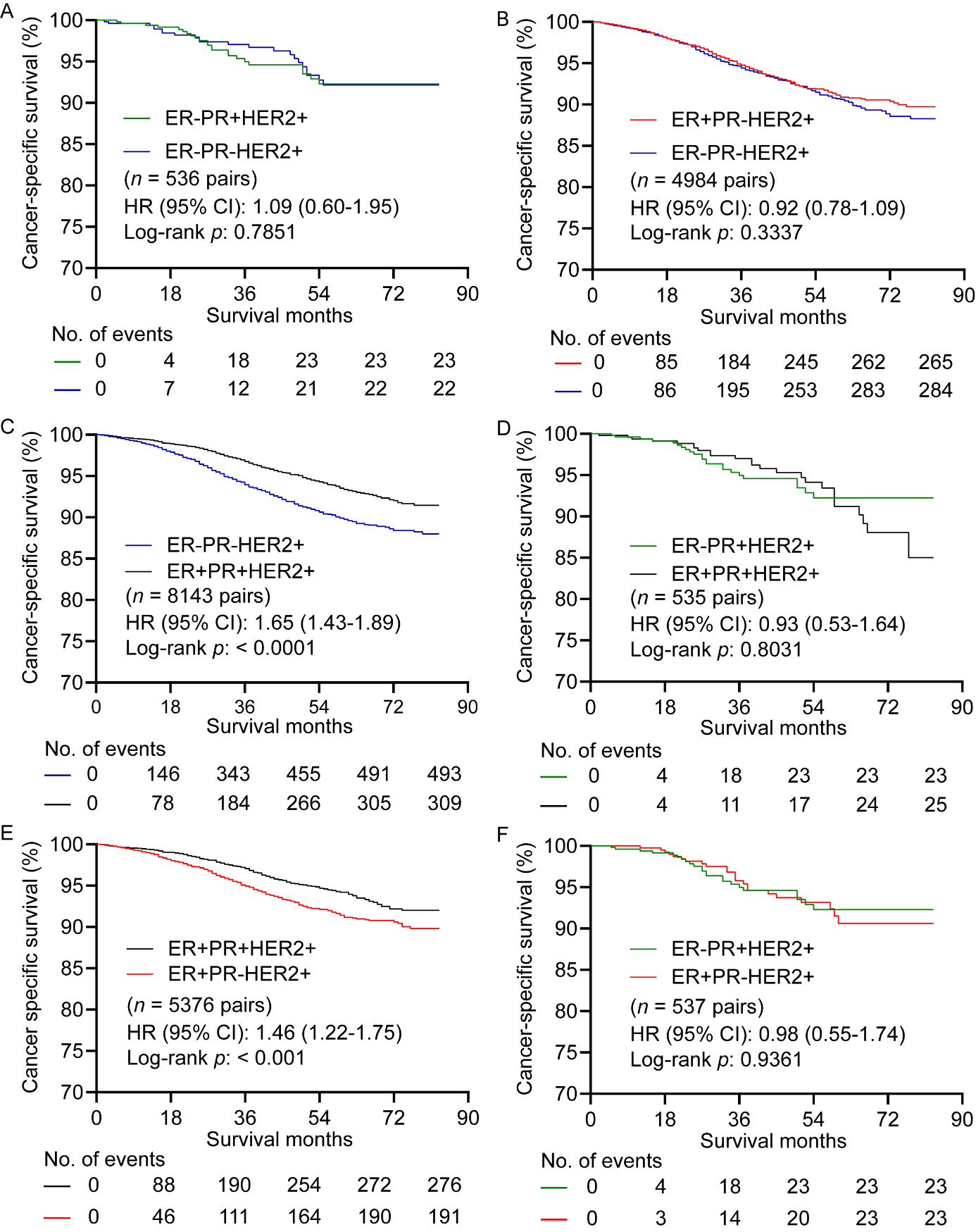


**Supplementary Fig. 4** Kaplan–Meier curves of breast cancer-specific survival in HER2+ subgroup after propensity score matching. (A) Comparison between ER-PR+ and ER-PR-. (B) Comparison between ER+PR- and ER-PR-. (C) Comparison between ER-PR- and ER+PR+. (D) Comparison between ER+PR+ and ER-PR+. (E) Comparison between ER+PR+ and ER+PR-. (F) Comparison between ER+PR- and ER-PR+. ER, estrogen receptor; PR, progesterone receptor; HER2, human epidermal growth factor Receptor 2.

| **Supplementary Table 1.** BCSS by multivariate Cox proportional analysis | | |
| --- | --- | --- |
| Category | HR (95% CI) | *P* value |
| Age | 1.017 (1.015-1.019) | < 0.001 |
| Year of diagnosis | 0.98 (0.97-0.99) | 0.013 |
| Race (vs White) |  |  |
| Black | 1.25 (1.17-1.32) | < 0.001 |
| Other | 0.77 (0.70-0.84) | < 0.001 |
| Insurance (vs uninsured) | |  |
| Insured | 0.84 (0.72-0.97) | 0.018 |
| Marital status (vs single) | |  |
| Married | 0.82 (0.77-0.87) | < 0.001 |
| Divorced/separated/widowed | 1.00 (0.94-1.08) | 0.932 |
| Other | 0.85 (0.75-0.96) | 0.009 |
| Grade (vs I) |  |  |
| II | 1.99 (1.77-2.24) | < 0.001 |
| III-IV | 3.68 (3.26-4.15) | < 0.001 |
| Pathology (vs invasive ductal carcinoma) | |  |
| Invasive lobular carcinoma | 0.94 (0.86-1.03) | 0.174 |
| IDLC | 0.99 (0.90-1.11) | 0.939 |
| Tumor (vs T1) |  |  |
| T2 | 2.14 (2.01-2.27) | < 0.001 |
| T3 | 3.32 (3.06-3.60) | < 0.001 |
| T4 | 4.15 (3.78-4.55) | < 0.001 |
| Node (vs N0) |  |  |
| N1 | 2.35 (2.21-2.50) | < 0.001 |
| N2 | 3.75 (3.46-4.07) | < 0.001 |
| N3 | 3.70 (3.31-4.14) | < 0.001 |
| Axillary lymph nodes metastasis | 1.04 (1.03-1.05) | < 0.001 |
| Metastasis (vs M0) |  |  |
| M1 | 3.44 (3.19-3.70) | < 0.001 |
| Molecular subtypes (vs ER+PR+HER2-) | |  |
| ER+PR-HER2- | 2.22 (2.06-2.39) | < 0.001 |
| ER-PR+HER2- | 3.07 (2.58-3.66) | < 0.001 |
| ER-PR-HER2- | 3.71 (3.48-3.95) | < 0.001 |
| ER+PR+HER2+ | 0.89 (0.80-0.99) | 0.026 |
| ER+PR-HER2+ | 1.40 (1.23-1.58) | < 0.001 |
| ER-PR+HER2+ | 1.08 (0.71-1.63) | 0.723 |
| ER-PR-HER2+ | 1.50 (1.36-1.65) | < 0.001 |
| Surgery (vs partial mastectomy) | |  |
| Total mastectomy | 1.08 (1.01-1.16) | 0.019 |
| Modified radical mastectomy | 1.22 (1.15-1.30) | < 0.001 |
| Radiation (vs no) |  |  |
| Yes | 0.73 (0.69-0.76) | < 0.001 |
| **Continuation** |  |  |
| Category | HR (95% CI) | *P* value |
| Chemotherapy (vs no) | |  |
| Yes | 0.82 (0.77-0.87) | < 0.001 |
| BCSS, breast cancer-specific survival; HR (95% CI), hazard ratio (95% confidence interval); IDLC, invasive ductal and lobular carcinoma; ER, estrogen receptor; PR, progesterone receptor; HER2, human epidermal growth factor receptor 2. | | |

| **Supplementary Table 2.** Comparison of the clinicopathologic characteristics between patients with ER-PR+HER2- and ER-PR-HER2- before and after PSM | | | | |
| --- | --- | --- | --- | --- |
| Category | ER-PR+HER2-  (*n* = 1395) | ER-PR-HER2-  (*n* = 1395) | *P*^1^ | *P*^2^ |
| Age | 57 (48-67) | 56 (47-66) | 0.420 | 0.002 |
| Year of diagnosis | 2013 (2011-2015) | 2013 (2011-2015) | 0.501 | 0.372 |
| Race |  |  |  |  |
| White | 1021 (73.2) | 1030 (73.8) | 0.866 | 0.556 |
| Black | 270 (19.4) | 259 (18.6) |  |  |
| Other | 104 (7.5) | 106 (7.6) |  |  |
| Insurance |  |  |  |  |
| Uninsured | 25 (1.8) | 19 (1.4) | 0.362 | 0.688 |
| Insured | 1370 (98.2) | 1376 (98.6) |  |  |
| Marital status |  |  |  |  |
| Single | 231 (16.6) | 230 (16.5) | 0.995 | 0.747 |
| Married | 796 (57.1) | 801 (57.4) |  |  |
| DSW | 308 (22.1) | 303 (21.7) |  |  |
| Other | 60 (4.3) | 61 (4.4) |  |  |
| Grade |  |  |  |  |
| I | 21 (1.5) | 17 (1.2) | 0.777 | 0.834 |
| II | 218 (15.6) | 224 (16.1) |  |  |
| III-IV | 1156 (82.9) | 1154 (82.7) |  |  |
| Pathology |  |  |  |  |
| IDC | 1350 (96.8) | 1353 (97.0) | 0.946 | 0.171 |
| ILC | 19 (1.4) | 18 (1.3) |  |  |
| IDLC | 26 (1.9) | 24 (1.7) |  |  |
| Stage |  |  |  |  |
| I | 531 (38.1) | 543 (38.9) | 0.099 | 0.012 |
| II | 681 (48.8) | 631 (45.2) |  |  |
| III | 161 (11.5) | 200 (14.3) |  |  |
| IV | 22 (1.6) | 21 (1.5) |  |  |
| Tumor |  |  |  |  |
| T1 | 633 (45.4) | 633 (45.4) | 0.155 | 0.307 |
| T2 | 621 (44.5) | 601 (43.1) |  |  |
| T3 | 89 (6.4) | 118 (8.5) |  |  |
| T4 | 52 (3.7) | 43 (3.1) |  |  |
| Node |  |  |  |  |
| N0 | 954 (68.4) | 973 (69.7) | 0.044 | 0.022 |
| N1 | 328 (23.5) | 280 (20.1) |  |  |
| N2 | 62 (4.4) | 86 (6.2) |  |  |
| N3 | 51 (3.7) | 56 (4.0) |  |  |
| ALNM | 1.04±3.39 | 1.09±3.46 | 0.725 | 0.092 |
| Metastasis |  |  |  |  |
| **Continuation** |  |  |  |  |
| Category | ER-PR+HER2-  (*n* = 1395) | ER-PR-HER2-  (*n* = 21439) | *P*^1^ | *P*^2^ |
| M0 | 1373 (98.4) | 1374 (98.5) | 0.878 | 0.392 |
| M1 | 22 (1.6) | 21 (1.5) |  |  |
| Surgery |  |  |  |  |
| Partial mastectomy | 785 (56.3) | 793 (56.8) | 0.926 | 0.027 |
| Total mastectomy | 370 (26.5) | 361 (25.9) |  |  |
| MRM | 240 (17.2) | 241 (17.3) |  |  |
| Radiation |  |  |  |  |
| No | 561 (40.2) | 532 (48.1) | 0.261 | 0.020 |
| Yes | 834 (59.8) | 863 (61.9) |  |  |
| Chemotherapy |  |  |  |  |
| No | 351 (25.2) | 348 (24.9) | 0.896 | 0.008 |
| Yes | 1044 (74.8) | 1047 (75.1) |  |  |
| *P*^1^ and *P*^2^ indicates comparison between ER-PR+HER2- and ER-PR-HER2- after and before PSM, respectively.  PSM was adjusted for age, year of diagnosis, ALNM, race, marital status, insurance, histology, grade, stage, tumor, node, metastasis, surgery, chemotherapy, and radiation.  Data were expressed as number (%), median (interquartile), or mean ± standard deviation.  ER, estrogen receptor; PR, progesterone receptor; HER2, human epidermal growth factor receptor 2; PSM, propensity score matching; DSW, divorced/separated/widowed; IDC, invasive ductal carcinoma; ILC, invasive lobular carcinoma; IDLC, invasive ductal and lobular carcinoma; ALNM, axillary lymph nodes metastasis; MRM, modified radical mastectomy | | | | |

| **Supplementary Table 3.** Comparison of the clinicopathologic characteristics between patients with ER+PR-HER2- and ER-PR-HER2- before and after PSM | | | | |
| --- | --- | --- | --- | --- |
| Category | ER+PR-HER2-  (*n* = 9626) | ER-PR-HER2-  (*n* = 9626) | *P*^1^ | *P*^2^ |
| Age | 59 (51-68) | 59 (49-68) | 0.013 | < 0.001 |
| Year of diagnosis | 2013 (2011-2015) | 2013 (2011-2015) | 0.799 | < 0.001 |
| Race |  |  |  |  |
| White | 7144 (74.2) | 6953 (72.2) | < 0.001 | < 0.001 |
| Black | 1515 (15.7) | 1886 (19.6) |  |  |
| Other | 967 (9.6) | 787 (8.2) |  |  |
| Insurance |  |  |  |  |
| Uninsured | 166 (1.7) | 161 (1.7) | 0.780 | < 0.001 |
| Insured | 9460 (98.7) | 9465 (98.3) |  |  |
| Marital status |  |  |  |  |
| Single | 1491 (15.5) | 1589 (16.5) | 0.051 | < 0.001 |
| Married | 5379 (55.9) | 5270 (54.7) |  |  |
| DSW | 2374 (24.7) | 2334 (24.2) |  |  |
| Other | 382 (4.0) | 433 (4.5) |  |  |
| Grade |  |  |  |  |
| I | 282 (2.9) | 308 (3.2) | 0.179 | < 0.001 |
| II | 3129 (32.5) | 3024 (31.4) |  |  |
| III-IV | 6215 (64.6) | 6294 (65.4) |  |  |
| Pathology |  |  |  |  |
| IDC | 9040 (93.9) | 9120 (94.6) | < 0.001 | < 0.001 |
| ILC | 459 (4.8) | 209 (2.2) |  |  |
| IDLC | 127 (1.3) | 297 (3.1) |  |  |
| Stage |  |  |  |  |
| I | 3809 (39.6) | 3928 (40.8) | 0.070 | < 0.001 |
| II | 4240 (44.0) | 4064 (42.2) |  |  |
| III | 1386 (14.4) | 1449 (15.1) |  |  |
| IV | 191 (2.0) | 185 (1.9) |  |  |
| Tumor |  |  |  |  |
| T1 | 4595 (47.7) | 4665 (48.5) | 0.341 | < 0.001 |
| T2 | 3992 (41.5) | 3875 (40.3) |  |  |
| T3 | 713 (7.4) | 741 (7.7) |  |  |
| T4 | 326 (3.4) | 345 (3.6) |  |  |
| Node |  |  |  |  |
| N0 | 6010 (62.4) | 6327 (65.7) | < 0.001 | 0.034 |
| N1 | 2542 (26.4) | 2162 (22.5) |  |  |
| N2 | 674 (7.0) | 682 (7.1) |  |  |
| N3 | 400 (4.2) | 455 (4.7) |  |  |
| ALNM | 1.34±3.48 | 1.29±3.45 | 0.296 | 0.839 |
| Metastasis |  |  |  |  |
| **Continuation** |  |  |  |  |
| Category | ER+PR-HER2-  (*n* = 9626) | ER-PR-HER2-  (*n* = 9626) | *P*^1^ | *P*^2^ |
| M0 | 9435 (98.0) | 9441 (98.1) | 0.755 | 0.004 |
| M1 | 191 (2.0) | 185 (1.9) |  |  |
| Surgery |  |  |  |  |
| Partial mastectomy | 5112 (53.1) | 5161 (53.6) | 0.663 | < 0.001 |
| Total mastectomy | 2539 (26.4) | 2485 (25.8) |  |  |
| MRM | 1975 (20.5) | 1980 (20.6) |  |  |
| Radiation |  |  |  |  |
| No | 4106 (42.7) | 4121 (42.8) | 0.827 | < 0.001 |
| Yes | 5520 (57.3) | 5505 (57.2) |  |  |
| Chemotherapy |  |  |  |  |
| No | 3152 (32.7) | 3033 (31.5) | 0.066 | < 0.001 |
| Yes | 6474 (67.3) | 6593 (68.5) |  |  |
| *P*^1^ and *P*^2^ indicates comparison between ER+PR-HER2- and ER-PR-HER2- after and before PSM, respectively.  PSM was adjusted for age, year of diagnosis, ALNM, race, marital status, insurance, histology, grade, stage, tumor, node, metastasis, surgery, chemotherapy, and radiation.  Data were expressed as number (%), median (interquartile), or mean ± standard deviation. ER, estrogen receptor; PR, progesterone receptor; HER2, human epidermal growth factor receptor 2; PSM, propensity score matching; DSW, divorced/separated/widowed; IDC, invasive ductal carcinoma; ILC, invasive lobular carcinoma; IDLC, invasive ductal and lobular carcinoma; ALNM, axillary lymph nodes metastasis; MRM, modified radical mastectomy | | | | |

| **Supplementary Table 4.** Comparison of the clinicopathologic characteristics between patients with ER-PR+HER2- and ER+PR-HER2- before and after PSM | | | | | | | | | |
| --- | --- | --- | --- | --- | --- | --- | --- | --- | --- |
| Category | | ER-PR+HER2-  (*n* = 1394) | | ER+PR-HER2-  (*n* = 1394) | | *P*^1^ | | *P*^2^ | |
| Age | | 55 (47-65) | | 56 (48-66) | | 0.241 | | < 0.001 | |
| Year of diagnosis | | 2013 (2011-2015) | | 2013 (2011-2015) | | 0.276 | | 0.330 | |
| Race | |  | |  | |  | |  | |
| White | | 1020 (73.2) | | 1045 (75.0) | | 0.055 | | < 0.001 | |
| Black | | 270 (19.4) | | 226 (16.2) | |  | |  | |
| Other | | 104 (7.5) | | 123 (8.8) | |  | |  | |
| Insurance | |  | |  | |  | |  | |
| Uninsured | | 25 (1.8) | | 23 (1.6) | | 0.771 | | 0.110 | |
| Insured | | 1369 (98.2) | | 1371 (98.4) | |  | |  | |
| Marital status | |  | |  | |  | |  | |
| Single | | 231 (16.6) | | 241 (17.3) | | 0.958 | | 0.006 | |
| Married | | 795 (57.1) | | 789 (56.6) | |  | |  | |
| DSW | | 308 (22.1) | | 307 (22.0) | |  | |  | |
| Other | | 60 (4.3) | | 57 (4.1) | |  | |  | |
| Grade | |  | |  | |  | |  | |
| I | | 21 (1.5) | | 24 (1.7) | | 0.817 | | < 0.001 | |
| II | | 218 (15.6) | | 209 (15.0) | |  | |  | |
| III-IV | | 1155 (82.9) | | 1161 (83.3) | |  | |  | |
| Pathology | |  | |  | |  | |  | |
| IDC | | 1349 (96.8) | | 1330 (95.4) | | 0.002 | | < 0.001 | |
| ILC | | 19 (1.4) | | 46 (3.3) | |  | |  | |
| IDLC | | 26 (1.9) | | 18 (1.3) | |  | |  | |
| Stage | |  | |  | |  | |  | |
| I | | 531 (38.1) | | 523 (37.5) | | 0.970 | | < 0.001 | |
| II | | 680 (48.8) | | 691 (49.6) | |  | |  | |
| III | | 161 (11.5) | | 160 (11.5) | |  | |  | |
| IV | | 22 (1.6) | | 20 (1.4) | |  | |  | |
| Tumor | |  | |  | |  | |  | |
| T1 | | 633 (45.4) | | 613 (44.0) | | 0.389 | | < 0.001 | |
| T2 | | 621 (44.5) | | 646 (46.3) | |  | |  | |
| T3 | | 88 (6.3) | | 96 (6.9) | |  | |  | |
| T4 | | 52 (3.7) | | 39 (2.8) | |  | |  | |
| Node | |  | |  | |  | |  | |
| N0 | | 953 (68.4) | | 909 (65.2) | | 0.261 | | 0.070 | |
| N1 | | 328 (23.5) | | 367 (26.3) | |  | |  | |
| N2 | | 62 (4.4) | | 71 (5.1) | |  | |  | |
| N3 | | 51 (3.7) | | 47 (3.4) | |  | |  | |
| ALNM | | 1.05±3.08 | | 1.10±3.16 | | 0.645 | | 0.112 | |
| Metastasis |  | |  | |  | |  | |  |
| **Continuation** | |  | |  | |  | |  | |
| Category | | ER-PR+HER2-  (*n* = 1394) | | ER+PR-HER2-  (*n* = 1394) | | *P*^1^ | | *P*^2^ | |
| M0 | | 1372 (98.4) | | 1374 (98.6) | | 0.756 | | 0.854 | |
| M1 | | 22 (1.6) | | 20 (1.4) | |  | |  | |
| Surgery | |  | |  | |  | |  | |
| Partial mastectomy | | 785 (56.3) | | 732 (52.5) | | 0.101 | | 0.242 | |
| Total mastectomy | | 369 (26.5) | | 388 (27.8) | |  | |  | |
| MRM | | 240 (17.2) | | 274 (19.7) | |  | |  | |
| Radiation | |  | |  | |  | |  | |
| No | | 560 (40.2) | | 585 (42.0) | | 0.336 | | 0.628 | |
| Yes | | 834 (59.8) | | 809 (58.0) | |  | |  | |
| Chemotherapy | |  | |  | |  | |  | |
| No | | 351 (25.2) | | 375 (26.9) | | 0.300 | | < 0.001 | |
| Yes | | 1043 (74.8) | | 1019 (73.1) | |  | |  | |
| *P*^1^ and *P*^2^ indicates comparison between ER-PR+HER2- and ER+PR-HER2- after and before PSM, respectively.  PSM was adjusted for age, year of diagnosis, ALNM, race, marital status, insurance, histology, grade, stage, tumor, node, metastasis, surgery, chemotherapy, and radiation.  Data were expressed as number (%), median (interquartile), or mean ± standard deviation. ER, estrogen receptor; PR, progesterone receptor; HER2, human epidermal growth factor receptor 2; PSM, propensity score matching; DSW, divorced/separated/widowed; IDC, invasive ductal carcinoma; ILC, invasive lobular carcinoma; IDLC, invasive ductal and lobular carcinoma; ALNM, axillary lymph nodes metastasis; MRM, modified radical mastectomy | | | | | | | | | |

| **Supplementary Table 5.** Comparison of the clinicopathologic characteristics between patients with ER+PR+HER2- and ER+PR-HER2- before and after PSM | | | | | | | | | |
| --- | --- | --- | --- | --- | --- | --- | --- | --- | --- |
| Category | | ER+PR-HER2-  (*n* = 16906) | | ER+PR+HER2-  (*n* = 16906) | | *P*^1^ | | *P*^2^ | |
| Age | | 62 (54-70) | | 62 (53-70) | | 0.241 | | < 0.001 | |
| Year of diagnosis | | 2013 (2011-2015) | | 2013 (2011-2015) | | 0.276 | | < 0.001 | |
| Race | |  | |  | |  | |  | |
| White | | 13095 (77.5) | | 13378 (79.1) | | < 0.001 | | < 0.001 | |
| Black | | 2196 (13.0) | | 1599 (9.5) | |  | |  | |
| Other | | 1615 (9.6) | | 1929 (11.4) | |  | |  | |
| Insurance | |  | |  | |  | |  | |
| Uninsured | | 217 (1.3) | | 216 (1.3) | | 0.961 | | 0.738 | |
| Insured | | 16689 (98.7) | | 16690 (98.7) | |  | |  | |
| Marital status | |  | |  | |  | |  | |
| Single | | 2413 (14.3) | | 2332 (13.8) | | 0.304 | | < 0.001 | |
| Married | | 9424 (55.7) | | 9586 (56.7) | |  | |  | |
| DSW | | 4369 (25.8) | | 4283 (25.3) | |  | |  | |
| Other | | 700 (4.1) | | 705 (4.2) | |  | |  | |
| Grade | |  | |  | |  | |  | |
| I | | 3415 (20.2) | | 2965 (17.5) | | < 0.001 | | < 0.001 | |
| II | | 7147 (42.3) | | 8259 (48.9) | |  | |  | |
| III-IV | | 6344 (37.5) | | 5682 (33.6) | |  | |  | |
| Pathology | |  | |  | |  | |  | |
| IDC | | 13397 (79.2) | | 13623 (80.6) | | < 0.001 | | < 0.001 | |
| ILC | | 2501 (14.8) | | 1950 (11.5) | |  | |  | |
| IDLC | | 1008 (6.0) | | 1333 (7.9) | |  | |  | |
| Stage | |  | |  | |  | |  | |
| I | | 8453 (50.0) | | 8353 (49.4) | | < 0.001 | | < 0.001 | |
| II | | 6107 (36.1) | | 6422 (38.0) | |  | |  | |
| III | | 2090 (12.4) | | 1863 (11.0) | |  | |  | |
| IV | | 256 (1.5) | | 268 (1.6) | |  | |  | |
| Tumor | |  | |  | |  | |  | |
| T1 | | 9704 (57.4) | | 9710 (57.4) | | 0.160 | | < 0.001 | |
| T2 | | 5663 (33.5) | | 5737 (33.9) | |  | |  | |
| T3 | | 1135 (6.7) | | 1038 (6.1) | |  | |  | |
| T4 | | 404 (2.4) | | 421 (2.5) | |  | |  | |
| Node | |  | |  | |  | |  | |
| N0 | | 11583 (68.5) | | 11274 (66.7) | | < 0.001 | | < 0.001 | |
| N1 | | 3682 (21.8) | | 4222 (25.0) | |  | |  | |
| N2 | | 1014 (6.0) | | 943 (5.6) | |  | |  | |
| N3 | | 627 (3.7) | | 467 (2.8) | |  | |  | |
| ALNM | | 1.18±3.38 | | 1.17±3.19 | | 0.694 | | < 0.001 | |
| Metastasis |  | |  | |  | |  | |  |
| **Continuation** | |  | |  | |  | |  | |
| Category | | ER+PR-HER2-  (*n* = 16906) | | ER+PR+HER2-  (*n* = 16906) | | *P*^1^ | | *P*^2^ | |
| M0 | | 16650 (98.5) | | 16638 (98.4) | | 0.597 | | < 0.001 | |
| M1 | | 256 (1.5) | | 268 (1.6) | |  | |  | |
| Surgery | |  | |  | |  | |  | |
| Partial mastectomy | | 9891 (58.5) | | 9939 (58.8) | | 0.661 | | < 0.001 | |
| Total mastectomy | | 4192 (24.8) | | 4206 (24.9) | |  | |  | |
| MRM | | 2823 (16.7) | | 2761 (16.3) | |  | |  | |
| Radiation | |  | |  | |  | |  | |
| No | | 6687 (39.6) | | 6699 (39.6) | | 0.894 | | < 0.001 | |
| Yes | | 10219 (60.4) | | 10207 (60.4) | |  | |  | |
| Chemotherapy | |  | |  | |  | |  | |
| No | | 9122 (54.0) | | 9115 (53.9) | | 0.939 | | < 0.001 | |
| Yes | | 7784 (46.0) | | 7791 (46.1) | |  | |  | |
| *P*^1^ and *P*^2^ indicates comparison between ER+PR+HER2- and ER+PR-HER2- after and before PSM, respectively.  PSM was adjusted for age, year of diagnosis, ALNM, race, marital status, insurance, histology, grade, stage, tumor, node, metastasis, surgery, chemotherapy, and radiation.  Data were expressed as number (%), median (interquartile), or mean ± standard deviation. ER, estrogen receptor; PR, progesterone receptor; HER2, human epidermal growth factor receptor 2; PSM, propensity score matching; DSW, divorced/separated/widowed; IDC, invasive ductal carcinoma; ILC, invasive lobular carcinoma; IDLC, invasive ductal and lobular carcinoma; ALNM, axillary lymph nodes metastasis; MRM, modified radical mastectomy | | | | | | | | | |

| **Supplementary Table 6.** Comparison of the clinicopathologic characteristics between patients with ER-PR+HER2- and ER+PR+HER2- before and after PSM | | | | | | | | | |
| --- | --- | --- | --- | --- | --- | --- | --- | --- | --- |
| Category | | ER-PR+HER2-  (*n* = 1395) | | ER+PR+HER2-  (*n* = 1395) | | *P*^1^ | | *P*^2^ | |
| Age | | 57 (48-67) | | 56 (48-64) | | 0.856 | | < 0.001 | |
| Year of diagnosis | | 2013 (2011-2015) | | 2013 (2011-2015) | | 0.614 | | 0.004 | |
| Race | |  | |  | |  | |  | |
| White | | 1021 (73.2) | | 1079 (77.3) | | < 0.001 | | < 0.001 | |
| Black | | 270 (19.4) | | 148 (10.6) | |  | |  | |
| Other | | 104 (7.5) | | 168 (12.0) | |  | |  | |
| Insurance | |  | |  | |  | |  | |
| Uninsured | | 25 (1.8) | | 22 (1.6) | | 0.659 | | 0.072 | |
| Insured | | 1370 (98.2) | | 1373 (98.4) | |  | |  | |
| Marital status | |  | |  | |  | |  | |
| Single | | 231 (16.6) | | 235 (16.8) | | 0.958 | | 0.048 | |
| Married | | 796 (57.1) | | 783 (56.1) | |  | |  | |
| DSW | | 308 (22.1) | | 313 (22.4) | |  | |  | |
| Other | | 60 (4.3) | | 64 (4.6) | |  | |  | |
| Grade | |  | |  | |  | |  | |
| I | | 21 (1.5) | | 49 (3.5) | | 0.003 | | < 0.001 | |
| II | | 218 (15.6) | | 201 (14.4) | |  | |  | |
| III-IV | | 1156 (82.9) | | 1145 (82.1) | |  | |  | |
| Pathology | |  | |  | |  | |  | |
| IDC | | 1350 (96.8) | | 1326 (95.1) | | 0.012 | | < 0.001 | |
| ILC | | 19 (1.4) | | 42 (3.0) | |  | |  | |
| IDLC | | 26 (1.9) | | 27 (1.9) | |  | |  | |
| Stage | |  | |  | |  | |  | |
| I | | 531 (38.1) | | 562 (40.3) | | 0.360 | | < 0.001 | |
| II | | 681 (48.8) | | 678 (48.6) | |  | |  | |
| III | | 161 (11.5) | | 136 (9.7) | |  | |  | |
| IV | | 22 (1.6) | | 19 (1.4) | |  | |  | |
| Tumor | |  | |  | |  | |  | |
| T1 | | 633 (45.4) | | 636 (45.6) | | 0.449 | | < 0.001 | |
| T2 | | 621 (44.5) | | 630 (45.2) | |  | |  | |
| T3 | | 89 (6.4) | | 92 (6.6) | |  | |  | |
| T4 | | 52 (3.7) | | 37 (2.7) | |  | |  | |
| Node | |  | |  | |  | |  | |
| N0 | | 954 (68.4) | | 966 (69.2) | | 0.018 | | 0.002 | |
| N1 | | 328 (23.5) | | 339 (24.3) | |  | |  | |
| N2 | | 62 (4.4) | | 66 (4.7) | |  | |  | |
| N3 | | 51 (3.7) | | 24 (1.7) | |  | |  | |
| ALNM | | 1.04±3.09 | | 0.98±2.78 | | 0.570 | | 0.145 | |
| Metastasis |  | |  | |  | |  | |  |
| **Continuation** | |  | |  | |  | |  | |
| Category | | ER-PR+HER2-  (*n* = 1395) | | ER+PR+HER2-  (*n* = 1395) | | *P*^1^ | | *P*^2^ | |
| M0 | | 1373 (98.4) | | 1376 (98.4) | | 0.637 | | 0.003 | |
| M1 | | 22 (1.6) | | 19 (1.4) | |  | |  | |
| Surgery | |  | |  | |  | |  | |
| Partial mastectomy | | 785 (56.3) | | 772 (55.3) | | 0.136 | | < 0.001 | |
| Total mastectomy | | 370 (26.5) | | 411 (29.5) | |  | |  | |
| MRM | | 240 (17.2) | | 212 (15.2) | |  | |  | |
| Radiation | |  | |  | |  | |  | |
| No | | 561 (40.2) | | 576 (41.3) | | 0.563 | | 0.035 | |
| Yes | | 834 (59.8) | | 819 (58.7) | |  | |  | |
| Chemotherapy | |  | |  | |  | |  | |
| No | | 351 (25.2) | | 365 (26.2) | | 0.544 | | < 0.001 | |
| Yes | | 1044 (74.8) | | 1030 (73.8) | |  | |  | |
| *P*^1^ and *P*^2^ indicates comparison between ER-PR+HER2- and ER+PR+HER2- after and before PSM, respectively.  PSM was adjusted for age, year of diagnosis, ALNM, race, marital status, insurance, histology, grade, stage, tumor, node, metastasis, surgery, chemotherapy, and radiation.  Data were expressed as number (%), median (interquartile), or mean ± standard deviation. ER, estrogen receptor; PR, progesterone receptor; HER2, human epidermal growth factor receptor 2; PSM, propensity score matching; DSW, divorced/separated/widowed; IDC, invasive ductal carcinoma; ILC, invasive lobular carcinoma; IDLC, invasive ductal and lobular carcinoma; ALNM, axillary lymph nodes metastasis; MRM, modified radical mastectomy | | | | | | | | | |

| **Supplementary Table 7.** Comparison of the clinicopathologic characteristics between patients with ER+PR+HER2- and ER+PR+HER2+ before and after PSM | | | | | | | | | |
| --- | --- | --- | --- | --- | --- | --- | --- | --- | --- |
| Category | | ER+PR+HER2+  (*n* = 15640) | | ER+PR+HER2-  (*n* = 15640) | | *P*^1^ | | *P*^2^ | |
| Age | | 56 (46-65) | | 56 (47-65) | | 0.740 | | < 0.001 | |
| Year of diagnosis | | 2013 (2011-2015) | | 2013 (2011-2015) | | 0.003 | | 0.002 | |
| Race | |  | |  | |  | |  | |
| White | | 12140 (77.6) | | 12149 (77.7) | | 0.201 | | < 0.001 | |
| Black | | 1735 (11.1) | | 1656 (10.6) | |  | |  | |
| Other | | 1765 (11.3) | | 1835 (11.7) | |  | |  | |
| Insurance | |  | |  | |  | |  | |
| Uninsured | | 263 (1.7) | | 280 (1.8) | | 0.462 | | < 0.001 | |
| Insured | | 15377 (98.3) | | 15360 (98.2) | |  | |  | |
| Marital status | |  | |  | |  | |  | |
| Single | | 2634 (16.8) | | 2544 (16.3) | | 0.316 | | < 0.001 | |
| Married | | 9295 (59.4) | | 9445 (60.4) | |  | |  | |
| DSW | | 3109 (19.9) | | 3042 (19.5) | |  | |  | |
| Other | | 602 (3.8) | | 609 (3.9) | |  | |  | |
| Grade | |  | |  | |  | |  | |
| I | | 1123 (7.2) | | 1300 (30.3) | | 0.001 | | < 0.001 | |
| II | | 6860 (43.9) | | 6749 (43.2) | |  | |  | |
| III-IV | | 7657 (49.0) | | 7591 (48.5) | |  | |  | |
| Pathology | |  | |  | |  | |  | |
| IDC | | 14216 (90.9) | | 13938 (89.1) | | < 0.001 | | < 0.001 | |
| ILC | | 657 (4.2) | | 1074 (6.9) | |  | |  | |
| IDLC | | 767 (4.9) | | 628 (4.0) | |  | |  | |
| Stage | |  | |  | |  | |  | |
| I | | 6909 (44.2) | | 6211 (39.7) | | < 0.001 | | < 0.001 | |
| II | | 6322 (40.4) | | 7167 (45.8) | |  | |  | |
| III | | 2124 (13.6) | | 1975 (12.6) | |  | |  | |
| IV | | 285 (1.8) | | 287 (1.8) | |  | |  | |
| Tumor | |  | |  | |  | |  | |
| T1 | | 8333 (53.3) | | 7923 (50.7) | | < 0.001 | | < 0.001 | |
| T2 | | 5879 (37.6) | | 6249 (40.0) | |  | |  | |
| T3 | | 968 (6.2) | | 1081 (6.9) | |  | |  | |
| T4 | | 460 (2.9) | | 387 (2.5) | |  | |  | |
| Node | |  | |  | |  | |  | |
| N0 | | 9795 (62.6) | | 8935 (57.1) | | < 0.001 | | < 0.001 | |
| N1 | | 4188 (26.8) | | 5172 (33.1) | |  | |  | |
| N2 | | 1087 (7.0) | | 1052 (6.7) | |  | |  | |
| N3 | | 570 (3.6) | | 481 (3.1) | |  | |  | |
| ALNM | | 1.24±3.18 | | 1.37±3.24 | | < 0.001 | | < 0.001 | |
| Metastasis |  | |  | |  | |  | |  |
| **Continuation** | |  | |  | |  | |  | |
| Category | | ER+PR+HER2+  (*n* = 15640) | | ER+PR+HER2-  (*n* = 15640) | | *P*^1^ | | *P*^2^ | |
| M0 | | 15355 (98.2) | | 15353 (98.2) | | 0.933 | | < 0.001 | |
| M1 | | 285 (1.8) | | 287 (1.8) | |  | |  | |
| Surgery | |  | |  | |  | |  | |
| Partial mastectomy | | 8043 (51.4) | | 7791 (49.8) | | 0.013 | | < 0.001 | |
| Total mastectomy | | 4604 (29.4) | | 4709 (30.1) | |  | |  | |
| MRM | | 2993 (19.1) | | 3140 (20.1) | |  | |  | |
| Radiation | |  | |  | |  | |  | |
| No | | 6903 (44.2) | | 6939 (44.4) | | 0. 682 | | < 0.001 | |
| Yes | | 8737 (55.8) | | 8701 (55.6) | |  | |  | |
| Chemotherapy | |  | |  | |  | |  | |
| No | | 3792 (24.2) | | 3725 (23.8) | | 0.375 | | < 0.001 | |
| Yes | | 11848 (75.8) | | 11915 (76.2) | |  | |  | |
| *P*^1^ and *P*^2^ indicates comparison between ER+PR+HER2+ and ER+PR+HER2- after and before PSM, respectively.  PSM was adjusted for age, year of diagnosis, ALNM, race, marital status, insurance, histology, grade, stage, tumor, node, metastasis, surgery, chemotherapy, and radiation.  Data were expressed as number (%), median (interquartile), or mean ± standard deviation. ER, estrogen receptor; PR, progesterone receptor; HER2, human epidermal growth factor receptor 2; PSM, propensity score matching; DSW, divorced/separated/widowed; IDC, invasive ductal carcinoma; ILC, invasive lobular carcinoma; IDLC, invasive ductal and lobular carcinoma; ALNM, axillary lymph nodes metastasis; MRM, modified radical mastectomy | | | | | | | | | |

| **Supplementary Table 8.** Comparison of the clinicopathologic characteristics between patients with ER-PR+HER2+ and ER-PR-HER2+ before and after PSM | | | | | | | | | |
| --- | --- | --- | --- | --- | --- | --- | --- | --- | --- |
| Category | | ER-PR+HER2+  (*n* = 536) | | ER-PR-HER2+  (*n* = 536) | | *P*^1^ | | *P*^2^ | |
| Age | | 56 (47-65) | | 55 (47-64) | | 0.550 | | 0.161 | |
| Year of diagnosis | | 2013 (2012-2015) | | 2013 (2011-2015) | | 0.497 | | 0.267 | |
| Race | |  | |  | |  | |  | |
| White | | 394 (73.5) | | 373 (69.6) | | 0.238 | | 0.659 | |
| Black | | 70 (13.1) | | 89 (16.6) | |  | |  | |
| Other | | 72 (13.4) | | 74 (13.8) | |  | |  | |
| Insurance | |  | |  | |  | |  | |
| Uninsured | | 10 (1.9) | | 11 (2.1) | | 0.826 | | 0.918 | |
| Insured | | 526 (98.1) | | 525 (97.9) | |  | |  | |
| Marital status | |  | |  | |  | |  | |
| Single | | 71 (13.2) | | 84 (15.7) | | 0.652 | | 0.568 | |
| Married | | 321 (59.9) | | 310 (57.8) | |  | |  | |
| DSW | | 118 (22.0) | | 120 (22.4) | |  | |  | |
| Other | | 26 (4.9) | | 22 (4.1) | |  | |  | |
| Grade | |  | |  | |  | |  | |
| I | | 6 (1.1) | | 4 (0.7) | | 0.674 | | 0.824 | |
| II | | 124 (23.1) | | 116 (21.6) | |  | |  | |
| III-IV | | 406 (75.7) | | 416 (77.6) | |  | |  | |
| Pathology | |  | |  | |  | |  | |
| IDC | | 522 (97.4) | | 519 (96.8) | | 0.227 | | 0.022 | |
| ILC | | 2 (0.4) | | 7 (1.3) | |  | |  | |
| IDLC | | 12 (2.2) | | 10 (1.9) | |  | |  | |
| Stage | |  | |  | |  | |  | |
| I | | 194 (36.2) | | 205 (38.2) | | 0.729 | | 0.282 | |
| II | | 243 (45.3) | | 234 (43.7) | |  | |  | |
| III | | 88 (16.4) | | 82 (15.3) | |  | |  | |
| IV | | 11 (2.1) | | 15 (2.8) | |  | |  | |
| Tumor | |  | |  | |  | |  | |
| T1 | | 252 (46.9) | | 249 (46.5) | | 0.748 | | 0.716 | |
| T2 | | 216 (40.3) | | 229 (42.7) | |  | |  | |
| T3 | | 40 (7.5) | | 35 (6.5) | |  | |  | |
| T4 | | 28 (5.2) | | 23 (4.3) | |  | |  | |
| Node | |  | |  | |  | |  | |
| N0 | | 319 (59.5) | | 344 (64.2) | | 0.339 | | 0.120 | |
| N1 | | 156 (29.1) | | 132 (24.6) | |  | |  | |
| N2 | | 42 (7.8) | | 38 (7.1) | |  | |  | |
| N3 | | 19 (3.5) | | 22 (4.1) | |  | |  | |
| ALNM | | 1.33±3.29 | | 1.21±3.21 | | 0.535 | | 0.206 | |
| Metastasis |  | |  | |  | |  | |  |
| **Continuation** | |  | |  | |  | |  | |
| Category | | ER-PR+HER2+  (*n* = 536) | | ER-PR-HER2+  (*n* = 536) | | *P*^1^ | | *P*^2^ | |
| M0 | | 525 (97.9) | | 521 (97.2) | | 0.427 | | 0.353 | |
| M1 | | 11 (2.1) | | 15 (2.8) | |  | |  | |
| Surgery | |  | |  | |  | |  | |
| Partial mastectomy | | 239 (44.6) | | 255 (47.6) | | 0.446 | | 0.224 | |
| Total mastectomy | | 180 (33.6) | | 161 (30.0) | |  | |  | |
| MRM | | 117 (21.8) | | 120 (22.4) | |  | |  | |
| Radiation | |  | |  | |  | |  | |
| No | | 245 (45.7) | | 239 (44.6) | | 0.713 | | 0.101 | |
| Yes | | 291 (54.3) | | 297 (55.4) | |  | |  | |
| Chemotherapy | |  | |  | |  | |  | |
| No | | 105 (19.6) | | 99 (18.5) | | 0.641 | | 0.441 | |
| Yes | | 431 (80.4) | | 437 (81.5) | |  | |  | |
| *P*^1^ and *P*^2^ indicates comparison between ER-PR+HER2+ and ER-PR-HER2+ after and before PSM, respectively.  PSM was adjusted for age, year of diagnosis, ALNM, race, marital status, insurance, histology, grade, stage, tumor, node, metastasis, surgery, chemotherapy, and radiation.  Data were expressed as number (%), median (interquartile), or mean ± standard deviation. ER, estrogen receptor; PR, progesterone receptor; HER2, human epidermal growth factor receptor 2; PSM, propensity score matching; DSW, divorced/separated/widowed; IDC, invasive ductal carcinoma; ILC, invasive lobular carcinoma; IDLC, invasive ductal and lobular carcinoma; ALNM, axillary lymph nodes metastasis; MRM, modified radical mastectomy | | | | | | | | | |

| **Supplementary Table 9.** Comparison of the clinicopathologic characteristics between patients with ER+PR-HER2+ and ER-PR-HER2+ before and after PSM | | | | | | | | | |
| --- | --- | --- | --- | --- | --- | --- | --- | --- | --- |
| Category | | ER+PR-HER2+  (*n* = 4984) | | ER-PR-HER2+  (*n* = 4984) | | *P*^1^ | | *P*^2^ | |
| Age | | 58 (50-66) | | 57 (50-66) | | 0.203 | | < 0.001 | |
| Year of diagnosis | | 2013 (2011-2015) | | 2013 (2011-2015) | | 0.950 | | 0.074 | |
| Race | |  | |  | |  | |  | |
| White | | 3733 (74.9) | | 3714 (74.5) | | 0.651 | | < 0.001 | |
| Black | | 615 (12.3) | | 645 (12.9) | |  | |  | |
| Other | | 636 (12.8) | | 625 (12.5) | |  | |  | |
| Insurance | |  | |  | |  | |  | |
| Uninsured | | 82 (1.6) | | 83 (1.7) | | 0.937 | | 0.467 | |
| Insured | | 4902 (98.4) | | 4901 (98.3) | |  | |  | |
| Marital status | |  | |  | |  | |  | |
| Single | | 787 (15.8) | | 726 (14.6) | | 0.187 | | 0.407 | |
| Married | | 2889 (58.0) | | 2989 (60.0) | |  | |  | |
| DSW | | 1102 (22.1) | | 1065 (21.4) | |  | |  | |
| Other | | 206 (4.1) | | 204 (4.1) | |  | |  | |
| Grade | |  | |  | |  | |  | |
| I | | 102 (2.0) | | 120 (2.4) | | 0.463 | | < 0.001 | |
| II | | 1763 (35.4) | | 1746 (35.0) | |  | |  | |
| III-IV | | 3119 (62.6) | | 3118 (62.6) | |  | |  | |
| Pathology | |  | |  | |  | |  | |
| IDC | | 4789 (96.1) | | 4822 (96.7) | | < 0.001 | | < 0.001 | |
| ILC | | 112 (2.2) | | 63 (1.3) | |  | |  | |
| IDLC | | 83 (1.7) | | 99 (2.0) | |  | |  | |
| Stage | |  | |  | |  | |  | |
| I | | 2095 (42.0) | | 2112 (42.4) | | 0.272 | | < 0.001 | |
| II | | 2043 (41.0) | | 1966 (39.4) | |  | |  | |
| III | | 711 (14.3) | | 770 (15.4) | |  | |  | |
| IV | | 135 (2.7) | | 136 (2.7) | |  | |  | |
| Tumor | |  | |  | |  | |  | |
| T1 | | 2558 (51.3) | | 2594 (52.0) | | 0.186 | | < 0.001 | |
| T2 | | 1883 (37.8) | | 1805 (36.2) | |  | |  | |
| T3 | | 375 (7.5) | | 385 (7.7) | |  | |  | |
| T4 | | 168 (3.4) | | 200 (4.0) | |  | |  | |
| Node | |  | |  | |  | |  | |
| N0 | | 3313 (62.5) | | 3127 (62.7) | | 0.654 | | < 0.001 | |
| N1 | | 1299 (26.1) | | 1256 (25.2) | |  | |  | |
| N2 | | 342 (6.9) | | 366 (7.3) | |  | |  | |
| N3 | | 230 (4.6) | | 235 (4.7) | |  | |  | |
| ALNM | | 1.26±3.22 | | 1.25±3.27 | | 0.929 | | < 0.001 | |
| Metastasis |  | |  | |  | |  | |  |
| **Continuation** | |  | |  | |  | |  | |
| Category | | ER+PR-HER2+  (*n* = 4984) | | ER-PR-HER2+  (*n* = 4984) | | *P*^1^ | | *P*^2^ | |
| M0 | | 4849 (97.3) | | 4848 (97.3) | | 0.951 | | 0.319 | |
| M1 | | 135 (2.7) | | 136 (2.7) | |  | |  | |
| Surgery | |  | |  | |  | |  | |
| Partial mastectomy | | 2256 (45.3) | | 2221 (44.6) | | 0.704 | | < 0.001 | |
| Total mastectomy | | 1612 (32.3) | | 1606 (32.4) | |  | |  | |
| MRM | | 1116 (22.4) | | 1147 (23.0) | |  | |  | |
| Radiation | |  | |  | |  | |  | |
| No | | 2442 (49.0) | | 2468 (49.5) | | 0.602 | | 0.276 | |
| Yes | | 2542 (51.0) | | 2516 (50.5) | |  | |  | |
| Chemotherapy | |  | |  | |  | |  | |
| No | | 1367 (23.4) | | 1137 (22.8) | | 0.476 | | < 0.001 | |
| Yes | | 3817 (76.6) | | 3847 (77.2) | |  | |  | |
| *P*^1^ and *P*^2^ indicates comparison between ER+PR-HER2+ and ER-PR-HER2+ after and before PSM, respectively.  PSM was adjusted for age, year of diagnosis, ALNM, race, marital status, insurance, histology, grade, stage, tumor, node, metastasis, surgery, chemotherapy, and radiation.  Data were expressed as number (%), median (interquartile), or mean ± standard deviation. ER, estrogen receptor; PR, progesterone receptor; HER2, human epidermal growth factor receptor 2; PSM, propensity score matching; DSW, divorced/separated/widowed; IDC, invasive ductal carcinoma; ILC, invasive lobular carcinoma; IDLC, invasive ductal and lobular carcinoma; ALNM, axillary lymph nodes metastasis; MRM, modified radical mastectomy | | | | | | | | | |

| **Supplementary Table 10.** Comparison of the clinicopathologic characteristics between patients with ER-PR-HER2+ and ER+PR+HER2+ before and after PSM | | | | | | | | | |
| --- | --- | --- | --- | --- | --- | --- | --- | --- | --- |
| Category | | ER-PR-HER2+  (*n* = 8143) | | ER+PR+HER2+  (*n* = 8143) | | *P*^1^ | | *P*^2^ | |
| Age | | 56 (48-65) | | 56 (46-66) | | 0.066 | | < 0.001 | |
| Year of diagnosis | | 2013 (2011-2015) | | 2013 (2011-2015) | | 0.905 | | < 0.001 | |
| Race | |  | |  | |  | |  | |
| White | | 5936 (72.9) | | 5993 (73.6) | | 0.032 | | < 0.001 | |
| Black | | 1132 (13.9) | | 1024 (12.6) | |  | |  | |
| Other | | 1075 (13.2) | | 1126 (13.8) | |  | |  | |
| Insurance | |  | |  | |  | |  | |
| Uninsured | | 150 (1.8) | | 145 (1.8) | | 0.769 | | 0.495 | |
| Insured | | 7993 (98.2) | | 7998 (98.2) | |  | |  | |
| Marital status | |  | |  | |  | |  | |
| Single | | 1264 (15.5) | | 1334 (16.4) | | 0.260 | | 0.001 | |
| Married | | 4860 (59.7) | | 4767 (58.5) | |  | |  | |
| DSW | | 1675 (20.6) | | 1719 (21.1) | |  | |  | |
| Other | | 344 (4.2) | | 323 (4.0) | |  | |  | |
| Grade | |  | |  | |  | |  | |
| I | | 120 (1.5) | | 109 (1.3) | | 0.757 | | < 0.001 | |
| II | | 1924 (23.6) | | 1919 (23.6) | |  | |  | |
| III-IV | | 6099 (74.9) | | 6115 (75.1) | |  | |  | |
| Pathology | |  | |  | |  | |  | |
| IDC | | 7974 (97.9) | | 7929 (97.4) | | 0.001 | | < 0.001 | |
| ILC | | 70 (0.9) | | 122 (1.5) | |  | |  | |
| IDLC | | 99 (1.2) | | 92 (1.1) | |  | |  | |
| Stage | |  | |  | |  | |  | |
| I | | 3182 (39.1) | | 3056 (37.5) | | 0.005 | | < 0.001 | |
| II | | 3342 (41.0) | | 3564 (43.8) | |  | |  | |
| III | | 1401 (17.2) | | 1313 (16.1) | |  | |  | |
| IV | | 218 (2.7) | | 210 (2.6) | |  | |  | |
| Tumor | |  | |  | |  | |  | |
| T1 | | 3950 (48.5) | | 3772 (46.3) | | < 0.001 | | < 0.001 | |
| T2 | | 3151 (38.7) | | 3491 (42.9) | |  | |  | |
| T3 | | 693 (8.5) | | 565 (6.9) | |  | |  | |
| T4 | | 349 (4.3) | | 315 (3.9) | |  | |  | |
| Node | |  | |  | |  | |  | |
| N0 | | 4950 (60.8) | | 4760 (58.5) | | < 0.001 | | < 0.001 | |
| N1 | | 2114 (26.0) | | 2336 (28.7) | |  | |  | |
| N2 | | 618 (7.6) | | 673 (8.3) | |  | |  | |
| N3 | | 461 (5.7) | | 374 (4.6) | |  | |  | |
| ALNM | | 1.47±3.83 | | 1.49±3.52 | | 0.748 | | < 0.001 | |
| Metastasis |  | |  | |  | |  | |  |
| **Continuation** | |  | |  | |  | |  | |
| Category | | ER-PR-HER2+  (*n* = 8143) | | ER+PR+HER2+  (*n* = 8143) | | *P*^1^ | | *P*^2^ | |
| M0 | | 7925 (97.3) | | 7933 (97.4) | | 0.695 | | < 0.001 | |
| M1 | | 218 (2.7) | | 210 (2.6) | |  | |  | |
| Surgery | |  | |  | |  | |  | |
| Partial mastectomy | | 3604 (44.3) | | 3682 (45.2) | | 0.115 | | < 0.001 | |
| Total mastectomy | | 2615 (32.1) | | 2492 (30.6) | |  | |  | |
| MRM | | 1924 (23.6) | | 1969 (24.2) | |  | |  | |
| Radiation | |  | |  | |  | |  | |
| No | | 3942 (48.4) | | 3927 (48.2) | | 0.814 | | < 0.001 | |
| Yes | | 4201 (51.6) | | 4216 (51.8) | |  | |  | |
| Chemotherapy | |  | |  | |  | |  | |
| No | | 1707 (21.0) | | 1686 (20.7) | | 0.685 | | < 0.001 | |
| Yes | | 6436 (79.0) | | 6457 (79.3) | |  | |  | |
| *P*^1^ and *P*^2^ indicates comparison between ER-PR-HER2+ and ER+PR+HER2+ after and before PSM, respectively.  PSM was adjusted for age, year of diagnosis, ALNM, race, marital status, insurance, histology, grade, stage, tumor, node, metastasis, surgery, chemotherapy, and radiation.  Data were expressed as number (%), median (interquartile), or mean ± standard deviation. ER, estrogen receptor; PR, progesterone receptor; HER2, human epidermal growth factor receptor 2; PSM, propensity score matching; DSW, divorced/separated/widowed; IDC, invasive ductal carcinoma; ILC, invasive lobular carcinoma; IDLC, invasive ductal and lobular carcinoma; ALNM, axillary lymph nodes metastasis; MRM, modified radical mastectomy | | | | | | | | | |

| **Supplementary Table 11.** Comparison of the clinicopathologic characteristics between patients with ER-PR+HER2+ and ER+PR+HER2+ before and after PSM | | | | | | | | | |
| --- | --- | --- | --- | --- | --- | --- | --- | --- | --- |
| Category | | ER-PR+HER2+  (*n* = 535) | | ER+PR+HER2+  (*n* = 535) | | *P*^1^ | | *P*^2^ | |
| Age | | 56 (47-65) | | 55 (46-65) | | 0.379 | | 0.660 | |
| Year of diagnosis | | 2013 (2012-2015) | | 2013 (2011-2015) | | 0.584 | | 0.429 | |
| Race | |  | |  | |  | |  | |
| White | | 394 (73.6) | | 407 (76.1) | | 0.656 | | 0.086 | |
| Black | | 70 (13.1) | | 63 (11.8) | |  | |  | |
| Other | | 71 (13.3) | | 65 (12.1) | |  | |  | |
| Insurance | |  | |  | |  | |  | |
| Uninsured | | 10 (1.9) | | 14 (2.6) | | 0.409 | | 0.748 | |
| Insured | | 525 (98.1) | | 521 (97.4) | |  | |  | |
| Marital status | |  | |  | |  | |  | |
| Single | | 71 (13.3) | | 83 (15.5) | | 0.710 | | 0.088 | |
| Married | | 322 (60.2) | | 307 (57.4) | |  | |  | |
| DSW | | 117 (21.9) | | 121 (22.6) | |  | |  | |
| Other | | 25 (4.7) | | 24 (4.5) | |  | |  | |
| Grade | |  | |  | |  | |  | |
| I | | 6 (1.1) | | 8 (1.5) | | 0.837 | | < 0.001 | |
| II | | 125 (23.4) | | 121 (22.6) | |  | |  | |
| III-IV | | 404 (75.5) | | 406 (75.9) | |  | |  | |
| Pathology | |  | |  | |  | |  | |
| IDC | | 520 (97.2) | | 517 (96.6) | | 0.075 | | < 0.001 | |
| ILC | | 2 (0.4) | | 9 (1.7) | |  | |  | |
| IDLC | | 13 (2.4) | | 9 (1.7) | |  | |  | |
| Stage | |  | |  | |  | |  | |
| I | | 194 (36.3) | | 182 (34.0) | | 0.872 | | 0.003 | |
| II | | 242 (45.2) | | 247 (46.2) | |  | |  | |
| III | | 87 (16.3) | | 94 (17.6) | |  | |  | |
| IV | | 12 (2.2) | | 12 (2.2) | |  | |  | |
| Tumor | |  | |  | |  | |  | |
| T1 | | 252 (47.1) | | 230 (43.0) | | 0.555 | | 0.002 | |
| T2 | | 216 (40.4) | | 233 (43.6) | |  | |  | |
| T3 | | 40 (7.5) | | 46 (8.6) | |  | |  | |
| T4 | | 27 (5.0) | | 26 (4.9) | |  | |  | |
| Node | |  | |  | |  | |  | |
| N0 | | 318 (59.4) | | 324 (60.6) | | 0.250 | | 0.504 | |
| N1 | | 156 (29.2) | | 137 (25.6) | |  | |  | |
| N2 | | 42 (7.9) | | 58 (10.8) | |  | |  | |
| N3 | | 19 (3.6) | | 16 (3.0) | |  | |  | |
| ALNM | | 1.33±3.29 | | 1.43±3.36 | | 0.633 | | 0.532 | |
| Metastasis |  | |  | |  | |  | |  |
| **Continuation** | |  | |  | |  | |  | |
| Category | | ER-PR+HER2+  (*n* = 535) | | ER+PR+HER2+  (*n* = 535) | | *P*^1^ | | *P*^2^ | |
| M0 | | 523 (97.8) | | 523 (97.8) | | 1.000 | | 0.498 | |
| M1 | | 12 (2.2) | | 12 (2.2) | |  | |  | |
| Surgery | |  | |  | |  | |  | |
| Partial mastectomy | | 240 (44.9) | | 251 (46.9) | | 0.414 | | 0.009 | |
| Total mastectomy | | 179 (33.5) | | 159 (29.7) | |  | |  | |
| MRM | | 116 (21.8) | | 125 (23.4) | |  | |  | |
| Radiation | |  | |  | |  | |  | |
| No | | 244 (45.6) | | 249 (46.5) | | 0.759 | | 0.449 | |
| Yes | | 291 (54.4) | | 286 (53.5) | |  | |  | |
| Chemotherapy | |  | |  | |  | |  | |
| No | | 105 (19.6) | | 102 (19.1) | | 0.816 | | 0.013 | |
| Yes | | 430 (80.4) | | 433 (80.9) | |  | |  | |
| *P*^1^ and *P*^2^ indicates comparison between ER-PR+HER2+ and ER+PR+HER2+ after and before PSM, respectively.  PSM was adjusted for age, year of diagnosis, ALNM, race, marital status, insurance, histology, grade, stage, tumor, node, metastasis, surgery, chemotherapy, and radiation.  Data were expressed as number (%), median (interquartile), or mean ± standard deviation. ER, estrogen receptor; PR, progesterone receptor; HER2, human epidermal growth factor receptor 2; PSM, propensity score matching; DSW, divorced/separated/widowed; IDC, invasive ductal carcinoma; ILC, invasive lobular carcinoma; IDLC, invasive ductal and lobular carcinoma; ALNM, axillary lymph nodes metastasis; MRM, modified radical mastectomy | | | | | | | | | |

| **Supplementary Table 12.** Comparison of the clinicopathologic characteristics between patients with ER+PR-HER2+ and ER+PR+HER2+ before and after PSM | | | | | | | | | |
| --- | --- | --- | --- | --- | --- | --- | --- | --- | --- |
| Category | | ER+PR-HER2+  (*n* = 5376) | | ER+PR+HER2+  (*n* = 5376) | | *P*^1^ | | *P*^2^ | |
| Age | | 58 (51-66) | | 59 (49-68) | | 0.962 | | < 0.001 | |
| Year of diagnosis | | 2013 (2011-2015) | | 2013 (2011-2015) | | 0.793 | | 0.001 | |
| Race | |  | |  | |  | |  | |
| White | | 4078 (75.9) | | 4067 (75.7) | | 0.917 | | 0.024 | |
| Black | | 644 (12.0) | | 658 (12.2) | |  | |  | |
| Other | | 654 (12.2) | | 651 (12.1) | |  | |  | |
| Insurance | |  | |  | |  | |  | |
| Uninsured | | 88 (1.6) | | 95 (1.8) | | 0.602 | | 0.822 | |
| Insured | | 5288 (98.4) | | 5281 (98.2) | |  | |  | |
| Marital status | |  | |  | |  | |  | |
| Single | | 837 (15.6) | | 825 (15.3) | | 0.969 | | < 0.001 | |
| Married | | 3122 (58.1) | | 3117 (58.0) | |  | |  | |
| DSW | | 1198 (22.3) | | 1217 (22.6) | |  | |  | |
| Other | | 219 (4.1) | | 217 (4.0) | |  | |  | |
| Grade | |  | |  | |  | |  | |
| I | | 236 (4.4) | | 234 (4.4) | | 0.680 | | < 0.001 | |
| II | | 1984 (36.9) | | 2028 (37.7) | |  | |  | |
| III-IV | | 3156 (58.7) | | 3114 (57.9) | |  | |  | |
| Pathology | |  | |  | |  | |  | |
| IDC | | 5010 (93.2) | | 4983 (92.7) | | 0.465 | | < 0.001 | |
| ILC | | 175 (3.3) | | 198 (3.7) | |  | |  | |
| IDLC | | 191 (3.6) | | 195 (3.6) | |  | |  | |
| Stage | |  | |  | |  | |  | |
| I | | 2324 (43.2) | | 2320 (43.2) | | 0.779 | | 0.003 | |
| II | | 2160 (40.2) | | 2191 (40.8) | |  | |  | |
| III | | 753 (14.0) | | 720 (13.4) | |  | |  | |
| IV | | 139 (2.6) | | 145 (2.7) | |  | |  | |
| Tumor | |  | |  | |  | |  | |
| T1 | | 2822 (52.5) | | 2777 (51.5) | | 0.033 | | 0.027 | |
| T2 | | 1991 (37.0) | | 2077 (38.6) | |  | |  | |
| T3 | | 388 (7.2) | | 326 (6.1) | |  | |  | |
| T4 | | 175 (3.3) | | 196 (3.6) | |  | |  | |
| Node | |  | |  | |  | |  | |
| N0 | | 3397 (62.3) | | 3332 (62.0) | | 0.015 | | 0.007 | |
| N1 | | 1370 (25.5) | | 1474 (27.4) | |  | |  | |
| N2 | | 363 (6.8) | | 375 (7.0) | |  | |  | |
| N3 | | 246 (4.6) | | 195 (3.6) | |  | |  | |
| ALNM | | 1.23±3.17 | | 1.22±3.00 | | 0.863 | | 0.843 | |
| Metastasis |  | |  | |  | |  | |  |
| **Continuation** | |  | |  | |  | |  | |
| Category | | ER+PR-HER2+  (*n* = 5376) | | ER+PR+HER2+  (*n* = 5376) | | *P*^1^ | | *P*^2^ | |
| M0 | | 5237 (97.4) | | 5231 (97.3) | | 0.718 | | < 0.001 | |
| M1 | | 139 (2.6) | | 145 (2.7) | |  | |  | |
| Surgery | |  | |  | |  | |  | |
| Partial mastectomy | | 2493 (46.4) | | 2560 (47.6) | | 0.074 | | < 0.001 | |
| Total mastectomy | | 1719 (32.0) | | 1610 (29.9) | |  | |  | |
| MRM | | 1164 (21.7) | | 1206 (22.4) | |  | |  | |
| Radiation | |  | |  | |  | |  | |
| No | | 2605 (48.5) | | 2564 (47.7) | | 0.429 | | < 0.001 | |
| Yes | | 2771 (51.5) | | 2812 (52.3) | |  | |  | |
| Chemotherapy | |  | |  | |  | |  | |
| No | | 1334 (24.8) | | 1323 (24.6) | | 0.806 | | 0.398 | |
| Yes | | 4042 (75.2) | | 4053 (75.4) | |  | |  | |
| *P*^1^ and *P*^2^ indicates comparison between ER+PR-HER2+ and ER+PR+HER2+ after and before PSM, respectively.  PSM was adjusted for age, year of diagnosis, ALNM, race, marital status, insurance, histology, grade, stage, tumor, node, metastasis, surgery, chemotherapy, and radiation.  Data were expressed as number (%), median (interquartile), or mean ± standard deviation. ER, estrogen receptor; PR, progesterone receptor; HER2, human epidermal growth factor receptor 2; PSM, propensity score matching; DSW, divorced/separated/widowed; IDC, invasive ductal carcinoma; ILC, invasive lobular carcinoma; IDLC, invasive ductal and lobular carcinoma; ALNM, axillary lymph nodes metastasis; MRM, modified radical mastectomy | | | | | | | | | |

| **Supplementary Table 13.** Comparison of the clinicopathologic characteristics between patients with ER-PR+HER2+ and ER+PR-HER2+ before and after PSM | | | | | | | | | |
| --- | --- | --- | --- | --- | --- | --- | --- | --- | --- |
| Category | | ER-PR+HER2+  (*n* = 537) | | ER+PR-HER2+  (*n* = 537) | | *P*^1^ | | *P*^2^ | |
| Age | | 56 (47-65) | | 58 (48-64) | | 0.921 | | < 0.001 | |
| Year of diagnosis | | 2013 (2012-2015) | | 2013 (2011-2015) | | 0.530 | | 0.675 | |
| Race | |  | |  | |  | |  | |
| White | | 395 (73.6) | | 410 (76.4) | | 0.354 | | 0.512 | |
| Black | | 70 (13.0) | | 55 (10.2) | |  | |  | |
| Other | | 72 (13.4) | | 72 (13.4) | |  | |  | |
| Insurance | |  | |  | |  | |  | |
| Uninsured | | 10 (1.9) | | 11 (2.0) | | 0.826 | | 0.695 | |
| Insured | | 527 (98.1) | | 526 (98.0) | |  | |  | |
| Marital status | |  | |  | |  | |  | |
| Single | | 71 (13.2) | | 86 (16.0) | | 0.345 | | 0.426 | |
| Married | | 322 (60.0) | | 294 (54.7) | |  | |  | |
| DSW | | 118 (22.0) | | 127 (23.6) | |  | |  | |
| Other | | 26 (4.8) | | 30 (5.6) | |  | |  | |
| Grade | |  | |  | |  | |  | |
| I | | 6 (1.1) | | 7 (1.3) | | 0.960 | | < 0.001 | |
| II | | 125 (23.3) | | 124 (23.1) | |  | |  | |
| III-IV | | 406 (75.6) | | 406 (75.6) | |  | |  | |
| Pathology | |  | |  | |  | |  | |
| IDC | | 522 (97.2) | | 519 (96.6) | | 0.700 | | < 0.001 | |
| ILC | | 2 (0.4) | | 4 (0.7) | |  | |  | |
| IDLC | | 13 (2.4) | | 14 (2.6) | |  | |  | |
| Stage | |  | |  | |  | |  | |
| I | | 194 (36.1) | | 202 (37.6) | | 0.909 | | 0.011 | |
| II | | 243 (45.3) | | 243 (45.3) | |  | |  | |
| III | | 88 (16.4) | | 80 (14.9) | |  | |  | |
| IV | | 12 (2.2) | | 12 (2.2) | |  | |  | |
| Tumor | |  | |  | |  | |  | |
| T1 | | 252 (46.9) | | 242 (45.1) | | 0.035 | | 0.020 | |
| T2 | | 217 (40.4) | | 216 (40.2) | |  | |  | |
| T3 | | 40 (7.4) | | 63 (11.7) | |  | |  | |
| T4 | | 28 (5.2) | | 16 (3.0) | |  | |  | |
| Node | |  | |  | |  | |  | |
| N0 | | 320 (59.6) | | 344 (64.1) | | 0.332 | | 0.146 | |
| N1 | | 156 (29.1) | | 139 (25.9) | |  | |  | |
| N2 | | 42 (7.8) | | 32 (6.0) | |  | |  | |
| N3 | | 19 (3.5) | | 22 (4.1) | |  | |  | |
| ALNM | | 1.33±3.29 | | 1.26±3.51 | | 0.733 | | 0.498 | |
| Metastasis |  | |  | |  | |  | |  |
| **Continuation** | |  | |  | |  | |  | |
| Category | | ER-PR+HER2+  (*n* = 537) | | ER+PR-HER2+  (*n* = 537) | | *P*^1^ | | *P*^2^ | |
| M0 | | 525 (97.8) | | 525 (97.8) | | 1.000 | | 0.575 | |
| M1 | | 12 (2.2) | | 12 (2.2) | |  | |  | |
| Surgery | |  | |  | |  | |  | |
| Partial mastectomy | | 240 (44.7) | | 242 (45.1) | | 0.335 | | 0.719 | |
| Total mastectomy | | 180 (33.5) | | 196 (36.5) | |  | |  | |
| MRM | | 117 (21.8) | | 99 (18.4) | |  | |  | |
| Radiation | |  | |  | |  | |  | |
| No | | 246 (45.8) | | 276 (51.4) | | 0.067 | | 0.234 | |
| Yes | | 291 (54.2) | | 261 (48.6) | |  | |  | |
| Chemotherapy | |  | |  | |  | |  | |
| No | | 105 (19.6) | | 103 (19.2) | | 0.877 | | 0.007 | |
| Yes | | 432 (80.4) | | 434 (80.8) | |  | |  | |
| *P*^1^ and *P*^2^ indicates comparison between ER-PR+HER2+ and ER+PR-HER2+ after and before PSM, respectively.  PSM was adjusted for age, year of diagnosis, ALNM, race, marital status, insurance, histology, grade, stage, tumor, node, metastasis, surgery, chemotherapy, and radiation.  Data were expressed as number (%), median (interquartile), or mean ± standard deviation. ER, estrogen receptor; PR, progesterone receptor; HER2, human epidermal growth factor receptor 2; PSM, propensity score matching; DSW, divorced/separated/widowed; IDC, invasive ductal carcinoma; ILC, invasive lobular carcinoma; IDLC, invasive ductal and lobular carcinoma; ALNM, axillary lymph nodes metastasis; MRM, modified radical mastectomy | | | | | | | | | |
